# Ambient oxygen enrichment during cardiopulmonary resuscitation: an overestimated risk? - Insights from a manikin study

**DOI:** 10.1186/s13049-026-01669-3

**Published:** 2026-08-11

**Authors:** Dominik J. Hoechter, Florian Pfeiffer, Michael Storz, Andreas Higl, Bjarne Schmalbach, Katja Petrowski, Tobias Achtsnit

**Affiliations:** 1 Department of Anaesthesiology, LMU University Hospital, LMU Munich, Marchioninistr. 15, 81377 Munich, Germany; 2 German Air Rescue Service Association “DRF Luftrettung”, Air Base Munich, Elisabeth-Stöber-Straße 71, D-81377 Munich, Germany; 3 City of Munich, Fire Department, Vocational School for Emergency Paramedics, An der Hauptfeuerwache 8, 80331 Munich, Germany; 4 City of Munich, Fire Department, Analytical Task Force & Center of Excellence for CBRN-Protection, An der Hauptfeuerwache 8, 80331 Munich, Germany; 5 Medical Psychology and Medical Sociology, University Medical Center of the Johannes Gutenberg University Mainz, Saarstraße 21 (Campus Universität), 55099 Mainz, Germany

**Keywords:** Cardiopulmonary resuscitation, CPR, Oxygen, Fire hazard, Defibrillation, Combustion

## Abstract

**Background/ aim:**

To reduce the risk of fire, the ERC resuscitation guidelines recommend removing all oxygen-delivery devices at least 1 m away from the patient’s chest prior to defibrillation. This recommendation is based on the assumption of relevant local oxygen accumulation. The presented study aimed to characterise ambient oxygen concentrations during cardiopulmonary resuscitation (CPR) and to assess the effect of removing oxygen-delivery devices.

**Methods:**

In a controlled mannequin-based simulation study, 20 resuscitation scenarios were performed under standardised conditions, varying oxygen delivery, airway management, ventilation mode, environment (room vs. ambulance), and distance of oxygen sources. Oxygen concentrations were continuously measured and recorded at 10-second intervals at three locations corresponding to typical defibrillation sites. Linear mixed-effects models were used to analyse predictors of oxygen fraction.

**Results:**

A total of 6,555 oxygen measurements were recorded. Median oxygen concentration was 21.6% (IQR 21.3–22.1%). Removing oxygen delivery devices to > 1 m did not result in lower oxygen concentrations compared with leaving them in proximity of the mannequin’s head. Endotracheal intubation compared to an open airway and the use of an OxyDemand valve compared to a reservoir with 15 L/min oxygen flow were associated with slightly lower oxygen fractions, whereas other factors (handling of upper garments, site, use of mechanical CPR) showed no clinically relevant effects.

**Conclusion:**

Clinically relevant oxygen accumulation at defibrillation sites was not observed. Likewise, removing oxygen-delivery devices did not result in a reduction in oxygen concentrations. Under the tested conditions, these findings do not support clinically relevant oxygen enrichment during CPR, questioning the risk of oxygen-enriched atmospheres during CPR as well as the role of routine removal of oxygen sources prior to defibrillation.

**Supplementary Information:**

The online version contains supplementary material available at 10.1186/s13049-026-01669-3.

## Background

Current resuscitation guidelines recommend ventilating the patient with the highest possible oxygen concentration during cardiopulmonary resuscitation [22]. Oxygen, however, is a key component of the “fire triad”, alongside fuel and an ignition source. Although not flammable itself, elevated oxygen concentrations lower ignition thresholds, accelerate combustion, and facilitate fire propagation [8]. In medical settings, oxygen concentrations greater than 24% are widely reported as a precautionary threshold to define an oxygen-enriched atmosphere and to indicate an increased potential fire hazard [9, 18, 19]. In oxygen-enriched environments, electrical arcing - for example, from electrosurgical units or poorly applied defibrillator paddles - may ignite surrounding materials and cause patient burns [3, 5, 17]. However, this threshold is primarily intended for hazard identification and prevention and does not represent a discrete concentration at which ignition or rapid combustion necessarily occurs. The actual combustion behaviour depends on the material, ignition source, and environmental conditions.

In oxygen-enriched atmospheres, clothing materials represent a direct hazard to personnel and patient safety. For cotton, flame propagation increases gradually with rising oxygen concentration up to approximately 30%, whereas a markedly accelerated increase in combustion has been reported at concentrations of approximately 30–35% and above [8].

Although self-adhesive defibrillation pads may reduce the likelihood of spark generation in comparison to paddles, the risk is not considered negligible. The European Resuscitation Council (ERC) Advanced Life Support (ALS) guidelines recommend specific precautions to minimise this risk by removing oxygen-delivery devices, such as masks, nasal cannulae, or bag-mask systems, and placing them at least 1 m away from the patient’s chest prior to defibrillation [21].

Data on oxygen distribution and its persistence in the vicinity of the thorax during resuscitation are scarce. The extent of local oxygen enrichment likely depends on the mode, flow, and direction of oxygen delivery, as well as surrounding air turbulence. Thus far, no systematic characterisation of these dependencies has been achieved. Brief interruptions of oxygen delivery immediately prior to defibrillation may be ineffective in case of relevant oxygen accumulation, while on the other hand, unnecessary in its absence [10].

Therefore, this study aimed to characterise oxygen concentrations in the vicinity of the thorax under different oxygen-delivery conditions and resuscitation setups, and to assess whether brief interruptions in oxygen administration meaningfully alter the defibrillation environment.

## Methods

We conducted a controlled mannequin-based simulation study. Ethical approval was waived by the institutional review board of the Medical Faculty of Ludwig Maximilian University of Munich (reference number: 25-0738-KB).

### Resuscitation scenario setup

The study was performed under standardised resuscitation conditions using a high-fidelity mannequin (Ambu^®^, Denmark) with a closed airway system to simulate realistic exhalation [1, 2]. Scenarios were conducted either in a closed indoor room without active mechanical ventilation or artificial air circulation, or in a stationary ambulance with all doors and windows closed and no active ventilation. Ambient temperature was monitored by the oxygen measurement devices and remained between 19 °C and 21 °C throughout the experiments. Ambient humidity was not recorded. Between scenarios, the room or ambulance was ventilated for several minutes by opening the windows or doors to allow sufficient air exchange. Each subsequent scenario was initiated only after the measured ambient oxygen concentration had returned to baseline.

**Fig. 1:**
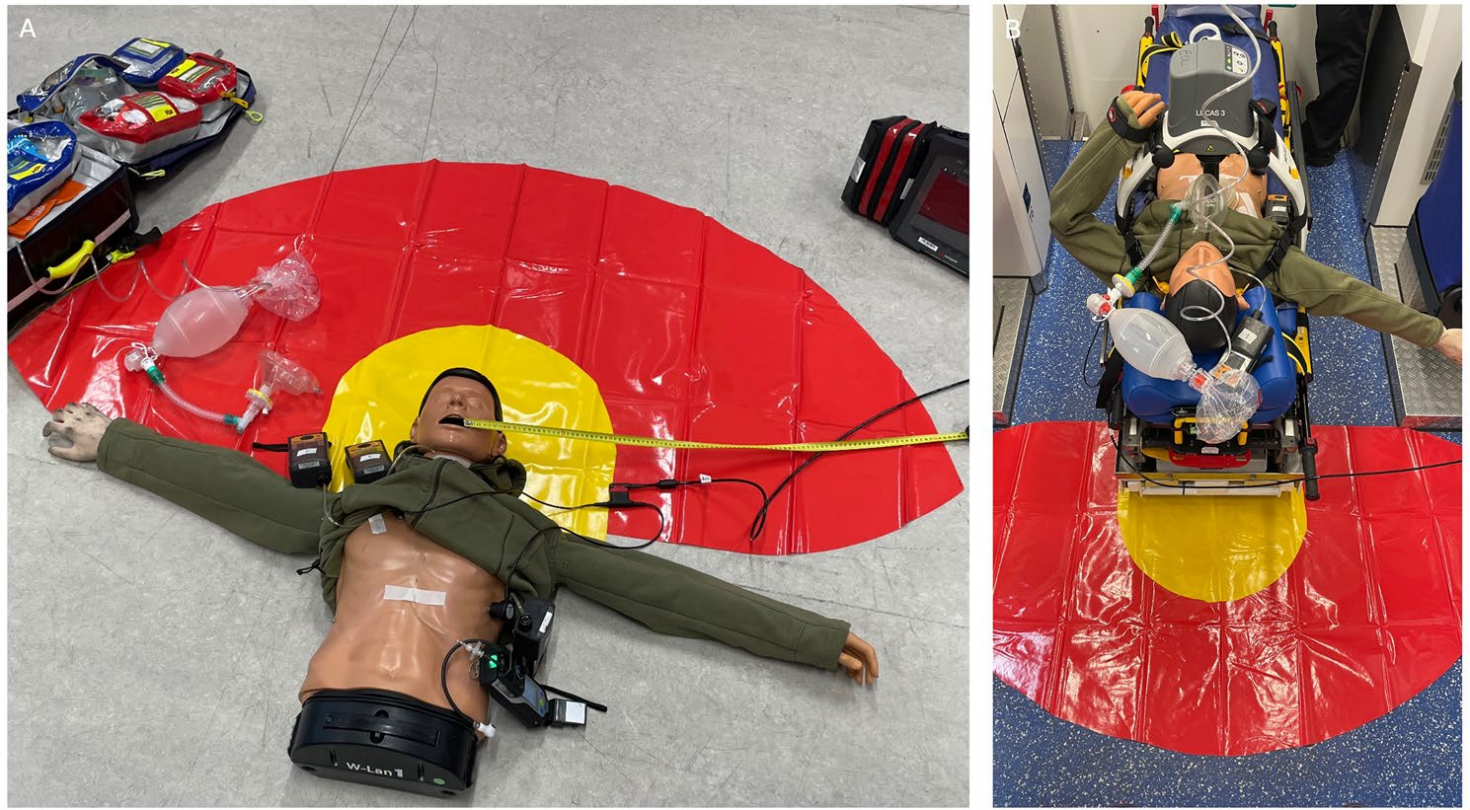
Set-up of the mannequin study: **A**: Set-up in the room, the yellow tarp indicates a radius of 0.2 m around the head, the red tarp indicates the distance of 1 m around the head. The sampling ports of the gas analysers were fixed to the respective measurement points using adhesive tape. A fourth gas analyser visible in the image at the level of the xiphoid process was not evaluated, as no changes in oxygen concentration were detected. During defibrillation, the ventilation bag was positioned either within the yellow sheet or outside the red sheet. Upper garments were either “lifted” - as shown in the figure - or “cut-open” by opening the zipper of the fleece jacket. **B**: experimental set-up in the ambulance with a mechanical CPR device in place

The mannequin was positioned supine with defibrillation pads applied in the antero-lateral position on the uncovered chest (Fig. 1A). The positions of the oxygen probes were secured by fixing the distal ends of the sampling tubes to the predefined anatomical landmarks using adhesive tape. Probe placement was checked regularly throughout the experiments. The experimental setup, probe locations, resuscitation sequence, oxygen-delivery conditions, and positioning of equipment were kept consistent unless deliberately varied as part of the respective scenario.

All iterations followed a standardised basic life support (BLS) scenario with a continuously shockable rhythm: After an initial 30 s of CPR with a 30:2 compression-to-ventilation ratio, rhythm analysis was performed, followed by defibrillator charging whilst continuing chest compressions, and shock delivery. CPR was then continued in 2-minute cycles. This sequence was repeated up to the fourth defibrillation, resulting in a total scenario duration of approximately 6.5–8 min. Each scenario was performed by exactly two ERC-certified ALS providers, providing chest compressions, ventilations, and defibrillations according to current ERC BLS and ALS guidelines. In scenarios with a secured airway, either via supraglottic airway (laryngeal mask) or endotracheal intubation, chest compressions were performed continuously with asynchronous ventilation.

Several forms of oxygen delivery, airway management, and ventilation were evaluated, including nasal cannula, non-rebreathing mask with reservoir, bag-valve-mask ventilation, and mechanical ventilation using two ventilators commonly employed in German emergency medical services (Medumat Standard², Weinmann, Germany, and Hamilton T1, Hamilton Medical, Switzerland).

The nasal cannula was operated at an oxygen flow rate of 6 L/min in accordance with its approved specifications, while the non-rebreathing oxygen mask was supplied with 15 L/min. In these scenarios, oxygen was administered continuously as passive oxygenation without additional ventilations, while chest compressions were performed continuously.

Bag-valve-mask ventilation was performed either at a compression-to-ventilation ratio of 30:2 or, in scenarios with continuous chest compressions and a secured airway, at a respiratory rate of 10 breaths/min. Ventilation was guided by visible chest rise, corresponding to an approximate tidal volume of 450 mL per breath. The bag-valve device was either operated with a reservoir at an oxygen flow rate of 15 L/min or connected to an oxygen-demand valve (Oxydemand^®^, DEHAS Medical Systems GmbH, Luebeck, Germany). Mechanical ventilation was performed in a volume-controlled mode with a tidal volume of 450 mL, a respiratory rate of 10 breaths/min, a positive end-expiratory pressure of 5 cmH₂O, and an inspired oxygen fraction of 1.0, in accordance with current resuscitation guidelines.

The airway was either left unprotected or secured using a second-generation supraglottic airway (laryngeal mask) or a cuffed endotracheal tube. From the onset of rhythm analysis until completion of defibrillation, oxygen delivery devices and ventilation bags were either removed and placed at a distance of more than 1 m from the patient’s chest, in accordance with current guidelines, or left in close proximity (within 20 cm of the mannequin’s head).

To account for potential confounding factors, additional experimental conditions were systematically varied. Chest compressions were performed either manually or using mechanical chest compression devices (LUCAS 3™, Stryker, or corpuls mCPR), as differences in provider movement may influence air turbulence and gas distribution. Furthermore, scenarios were conducted both in a standard indoor environment and in a stationary ambulance with closed doors and no active air exchange (Fig. 1B). This approach was chosen to evaluate whether a confined space may affect local oxygen accumulation. Finally, the influence of upper-body garments was assessed by either lifting the clothing to the upper chest or cutting it open to allow lateral displacement. All scenarios are listed in Table 1 (Table 1). Scenarios and repeated runs were performed sequentially in a predefined, non-randomised order.

### Oxygen measurements

Oxygen concentrations were measured continuously with measurements recorded at 10-second intervals using calibrated handheld gas monitors (Thermo Scientific MX6 iBrid^®^ with an electrochemical oxygen sensor (range: 0–30 Vol. %, resolution: 0.1)) at three predefined locations: (1) ventral neck, (2) right infraclavicular region, and (3) left lateral thoracic wall (axillary line), corresponding to typical defibrillation pad positions. Measurements were recorded at 10-second intervals. To ensure consistent sensor positioning, each sensor was connected to a 15-cm flexible sampling tube fixed at the respective measurement site.

### Reproducibility

Initially, scenarios were performed in triplicate. A variance component analysis of the repeated runs yielded an intraclass correlation coefficient of 0.04 for runs nested within scenarios, indicating that only a small proportion of the total variability was attributable to systematic differences between runs within the same scenario. Based on this low between-run variability, subsequent scenarios were conducted in duplicate.

### Statistical analyses

Oxygen concentrations were analysed descriptively and are presented as median and interquartile range (IQR), along with minimum and maximum values. Oxygen fraction was analysed using linear mixed-effects models with a random intercept for scenario and run (id) to account for repeated measurements. Models were fitted by restricted maximum likelihood, with Satterthwaite’s approximation used to estimate degrees of freedom. Univariate models were constructed for each predictor. Runtime was included as a continuous fixed effect in all models, and interaction terms between runtime and each predictor were added to assess time-dependent effects. To evaluate the influence of airway type, oxygen delivery method during manual ventilation, and handling of upper garments, predefined scenario sets were compared. Fixed effects were evaluated using Type III analysis of variance. Model performance was described using marginal and conditional R². Statistical analyses were performed in R (R Foundation for Statistical Computing, Vienna, Austria) using the lme4 and lmerTest, and MuMIn packages. A two-sided p-value < 0.05 was considered statistically significant.

**Table 1:** Main characteristics of all study scenarios. Scenarios were conducted either in a room without artificial ventilation or in a stationary ambulance without air conditioning or other forms of artificial air movement. The ventilation bag was connected either to a reservoir supplied with oxygen at 15 L/min or to an oxygen demand valve. “Distance” refers to the distance between the oxygen source or ventilation bag and the mannequin’s head during defibrillation. The oxygen mask and nasal cannula were either left in place on the mannequin’s face or removed to a distance greater than 1 m

	Site	Airway	Ventilation	Oxygen	Upper garments	Chest compressions	Distance
Scenario 1	Room	Open	Bag-valve-mask	Reservoir	Lifted	Manually	> 1 m
Scenario 2	Room	Open	Bag-valve-mask	Reservoir	Lifted	Manually	< 0.2 m
Scenario 3	Room	Open	Bag-valve-mask	Reservoir	Lifted	mCPR - LUCAS 3 ^®^	< 0.2 m
Scenario 4	Room	Open	Bag-valve-mask	Demand ^®^	Lifted	mCPR - LUCAS 3 ^®^	< 0.2 m
Scenario 5	Ambulance	Open	Bag-valve-mask	Reservoir	Lifted	mCPR - LUCAS 3 ^®^	< 0.2 m
Scenario 6	Ambulance	Open	Bag-valve-mask	Reservoir	Lifted	mCPR - LUCAS 3 ^®^	> 1 m
Scenario 7	Ambulance	Open	Bag-valve-mask	Demand ^®^	Lifted	mCPR - LUCAS 3 ^®^	< 0.2 m
Scenario 8	Ambulance	Endotracheal tube	Bag-valve-mask	Demand ^®^	Lifted	mCPR - LUCAS 3 ^®^	< 0.2 m
Scenario 9	Ambulance	Endotracheal tube	Weinmann respirator	FiO2 100%	Lifted	mCPR - LUCAS 3 ^®^	No disconnection of respirator
Scenario 10	Ambulance	Endotracheal tube	Weinmann respirator	FiO2 100%	Lifted	mCPR - LUCAS 3 ^®^	Disconnection and pause of ventilator
Scenario 11	Ambulance	Endotracheal tube	Hamilton T1	FiO2 100%	Lifted	mCPR - LUCAS 3 ^®^	Disconnection and pause of ventilator
Scenario 12	Ambulance	Endotracheal tube	Hamilton T1	FiO2 100%	Lifted	mCPR - LUCAS 3 ^®^	No disconnection of respirator
Scenario 13	Ambulance	Laryngeal mask	Bag-valve-mask	Demand ^®^	Lifted	mCPR - LUCAS 3 ^®^	< 0.2 m
Scenario 14	Ambulance	Laryngeal mask	Bag-valve-mask	Reservoir	Lifted	mCPR - LUCAS 3 ^®^	< 0.2 m
Scenario 15	Ambulance	Open	Bag-valve-mask	Reservoir	Cut open	mCPR - LUCAS 3 ^®^	< 0.2 m
Scenario 16	Ambulance	Open	Bag-valve-mask	Demand ^®^	Cut open	mCPR - LUCAS 3 ^®^	< 0.2 m
Scenario 17	Room	Open	Oxygen mask	15 L/min	Cut open	mCPR - corpulsCPR	Left in place
Scenario 18	Room	Open	Oxygen mask	15 L/min	Cut open	mCPR - corpulsCPR	> 1 m
Scenario 19	Room	Open	Nasal cannula	6 L/min	Cut open	mCPR - corpulsCPR	Left in place
Scenario 20	Room	Open	Nasal cannula	6 L/min	Cut open	mCPR - corpulsCPR	> 1 m

## Results

A total of 20 experimental scenarios were conducted. Of these, four scenarios were performed in triplicate and the remaining 16 scenarios in duplicate, resulting in a total of 65 individual experimental runs. Across all runs and measurement sites, a total of 6,555 oxygen concentration measurements were recorded.

**Fig. 2:**
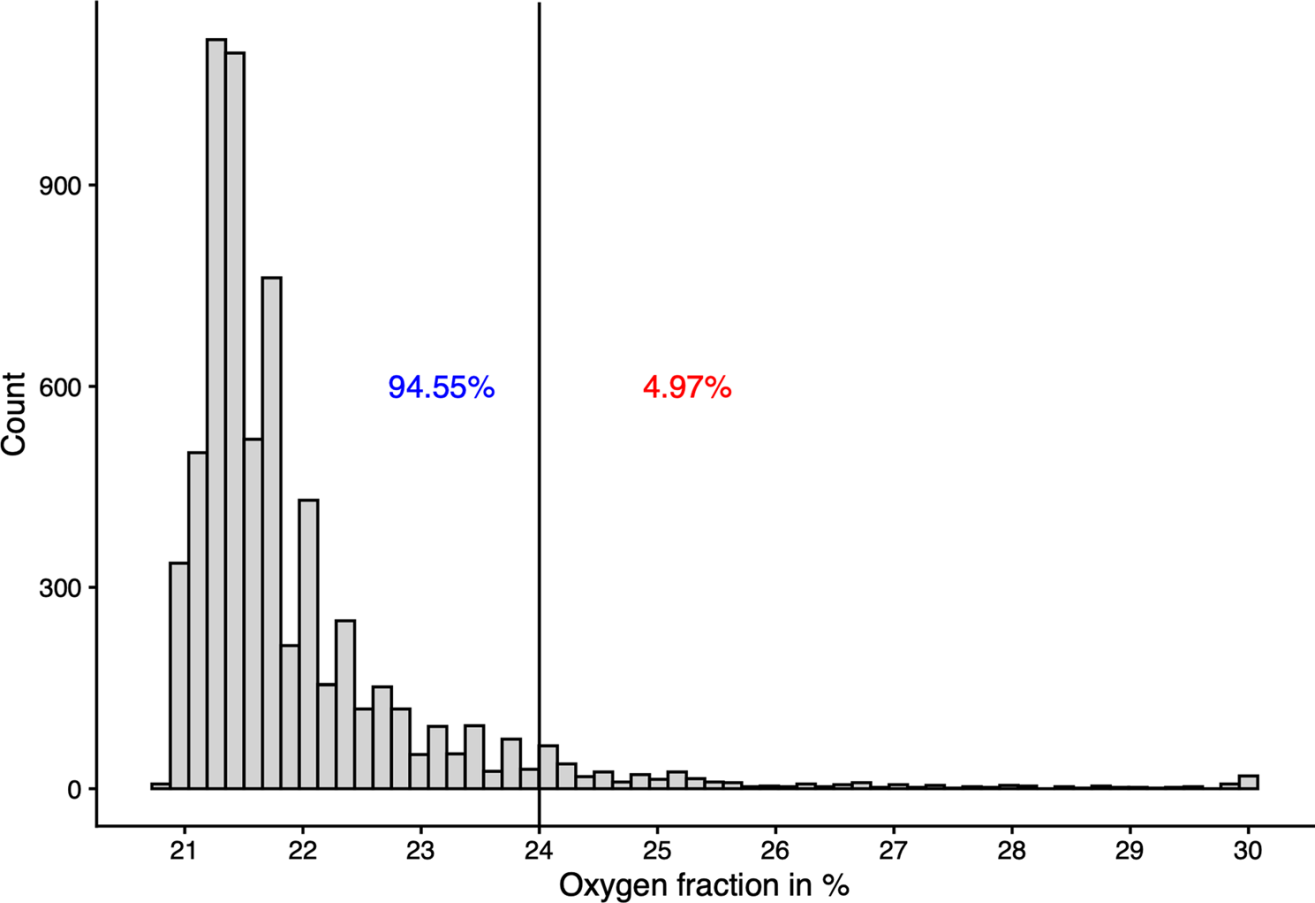
Histogram showing the distribution of measured oxygen concentrations across all experimental runs and measurement sites: 95% of values were below an oxygen fraction of 24%. The 24% threshold is shown as a reference value, as oxygen concentrations above this level are commonly regarded as associated with increased combustion risk

**Fig. 3:**
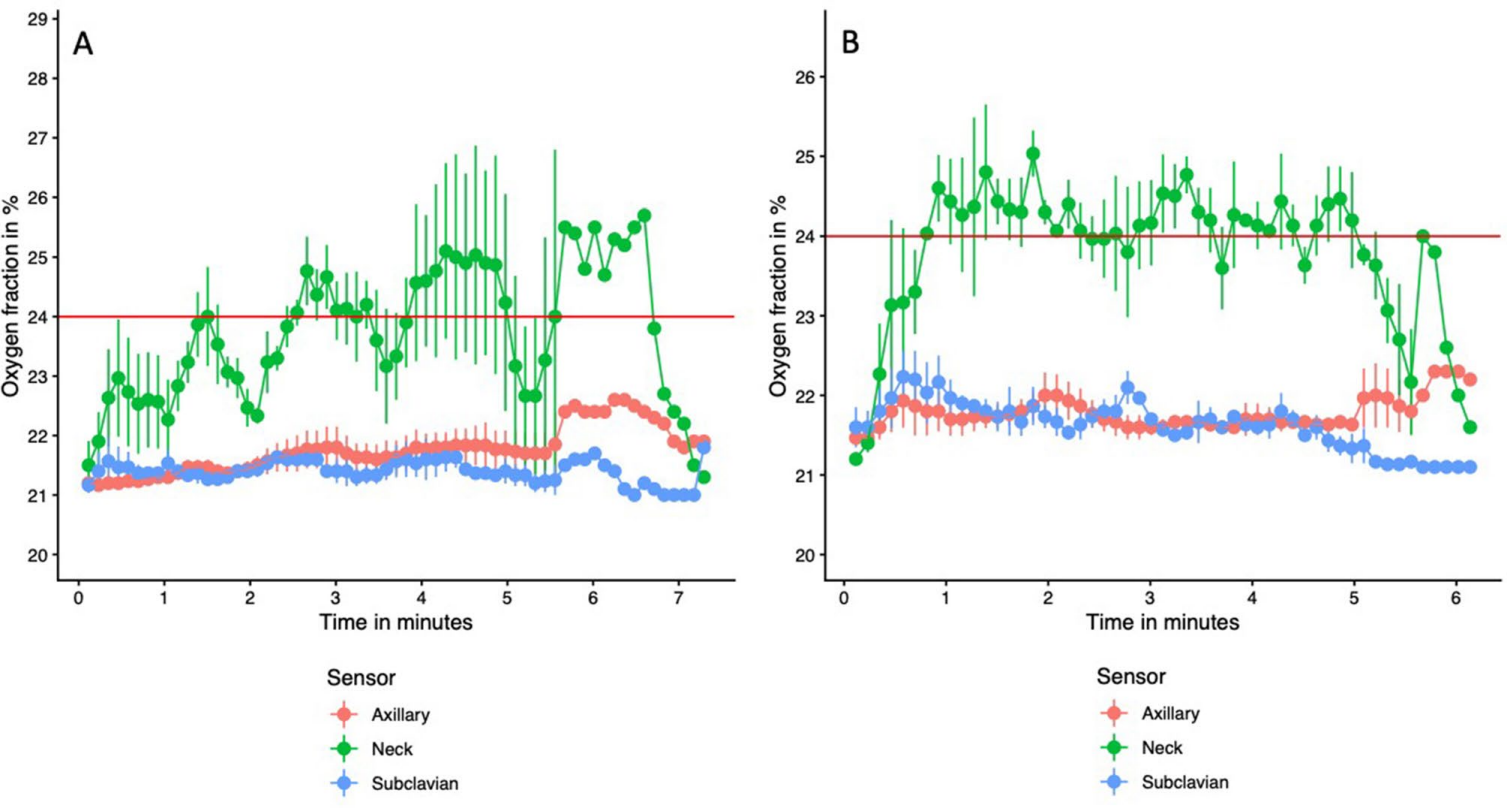
Time-dependent oxygen concentration profiles. Oxygen concentrations are shown over time for the three predefined measurement sites: ventral neck, right subclavian, and left axillary. Panel **A** depicts measurements for scenario 1, an advanced life support scenario in which the ventilation bag is placed > 1 m from the patient’s head during defibrillation. Panel **B** depicts measurements for scenario 2, the otherwise identical scenario in which the ventilation bag is positioned close to the patient’s head (< 0.2 m) during defibrillation. Time-oxygen concentration profiles of all scenarios are depicted in Supplement A

Overall, oxygen concentrations across all scenarios and measurement sites had a median of 21.6% (IQR 21.3–22.1%). Most oxygen fraction measurements were below 24% (95%), while only 5% exceeded this threshold (Fig. 2). At the ventral neck, the median was 21.2% (IQR 21.2–21.5%), with values ranging from 20.9% to 30.0%. At the right infraclavicular region, the median was 21.2% (IQR 21.2–21.4%), with a range of 21.0% to 24.0%. In the left axilla, the median was 21.2% (IQR 21.2–21.3%), with values ranging from 20.9% to 23.5%. (Figure 3, all plots available in Supplement A). No marked increases in oxygen concentration were observed during ventilation periods, nor were pronounced decreases detected when the oxygen source was removed from the patient.

**Fig. 4:**
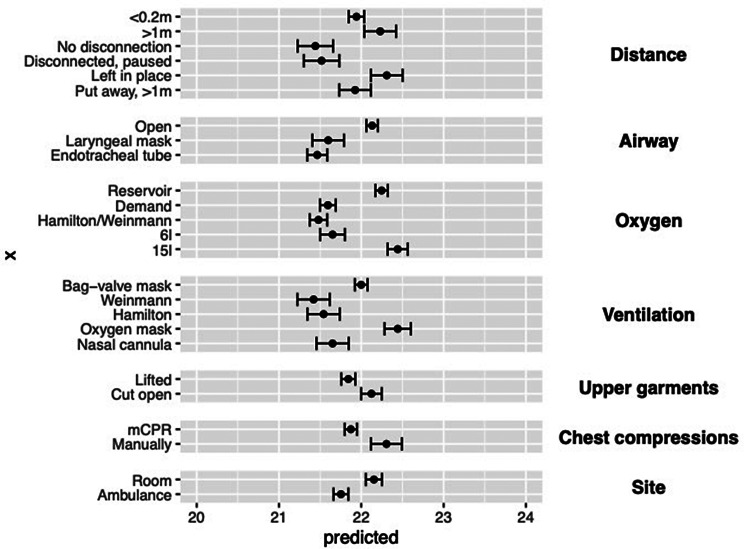
Estimated marginal means and confidence intervals for factors associated with oxygen concentration. The plot shows model-based estimated marginal means of oxygen concentration across predefined experimental conditions; see also Table 2

Across all scenarios, the oxygen fraction increased significantly over their respective runtime (all *p* < 0.001), although the effect was very small (R2m = 0.012). In predefined contrast analyses, the effects of selected experimental conditions on oxygen concentrations were assessed. Neither moving the ventilator bag more than 1 m away from the patient’s head nor disconnecting and pausing the ventilator significantly affected oxygen concentrations (*p* = 0.758 and *p* = 0.830). Further, lifting or cutting of the upper garments had no significant effect (*p* = 0.801). In addition, no significant effect was observed for the use of a mechanical CPR device (*p* = 0,084). Oxygen concentrations were likewise unaffected by the experimental setting, whether scenarios were performed inside an ambulance or in a room (*p* = 0.335). When assessing the influence of the airway, lower oxygen fraction values were observed for the endotracheal tube (β = −0.65) and the laryngeal mask (β = −0.52) compared with an open airway (*p* = 0.004). Similarly, the use of an oxygen demand-valve compared to the use of a reservoir bag with 15 L/min oxygen flow was associated with lower oxygen fractions (β = −0.46, *p* = 0.014), with a less pronounced increase over time (interaction β = −0.065, *p* < 0.001; R2m = 0.11)., with no evidence of time-dependent effects (Table 2; Fig. 4).

**Table 2:** Univariate mixed-effects analyses of predictors of oxygen fraction. For each factor, the main effect and its interaction with runtime were tested. Results are presented as F-values with corresponding degrees of freedom (df1, df2), p-values, and marginal R². Interaction terms indicate changes in the association between the predictor and oxygen fraction over time

Predictor	F	df1	df2	*p*	*R* ^2^
Distance	0.100	1	9.254	0.758	0.013
Distance X runtime	9.723	1	3,467.903	0.002	0.019
Connection to ventilator	0.058	1	2.260	0.830	0.017
Connection to ventilator X runtime	17.858	1	1,131.290	0.000	0.257
Upper garments	0.082	1	2.044	0.801	0.014
Upper garments X runtime	0.104	1	1,104.829	0.747	0.049
Demand valve	8.187	1	11.894	0.014	0.111
Demand valve X runtime	13.617	1	3,736.023	0.000	0.018
Airway	8.283	2	14.944	0.004	0.131
Airway X runtime	0.039	2	4,869.896	0.962	0.022
Site	1.058	1	7.590	0.335	0.002
sIte X runtime	13.108	1	2,891.855	0.000	0.017
Compressions	3.356	1	17.959	0.084	0.017
Compressions X runtime	3.063	1	6,532.884	0.080	0.012

## Discussion

In this manikin-based study, no clinically relevant oxygen accumulation was observed at the defibrillation site, even under deliberately unfavourable conditions with minimised air turbulence. Measured oxygen concentrations at the anterior chest wall remained low, with maximum values not exceeding 24%. These findings challenge the assumption that oxygen enrichment in the vicinity of defibrillation electrodes represents a major hazard during cardiopulmonary resuscitation.

Similar experimental studies have reported localised increases in oxygen concentration, particularly in the left axillary region [4, 20]. However, interpretation of these findings is limited by the characteristics of the manikin models used: LittleAnne^®^ manikins (Laerdal, Norway) exhibit non-physiological airflow behaviour, as one-way valve mechanisms prevent exhaled gas from exiting via the upper airway (personal communication with a Laerdal sales representative). Instead, insufflated air is diverted into internal compartments, creating artificial and potentially misleading patterns of gas leakage. In contrast, the AmbuMan^®^ (Ambu, Denmark) manikin used in the present study allowed exhalation through the natural airway, more closely mimicking physiological conditions. Interestingly, we observed similar localised oxygen accumulation in the left axillary region, namely when a defect in the lung system was present (Supplement B). This supports the hypothesis that previously described oxygen accumulations may at least partially reflect mannequin-specific artefacts rather than clinically relevant phenomena.

Furthermore, it is noteworthy that the above-mentioned studies demonstrating limited and highly localised oxygen enrichment have led to conflicting clinical interpretations. While some authors advocated for routine removal of oxygen sources prior to defibrillation as a simple precautionary measure, others have argued that the potential delay in defibrillation and interruption of oxygen delivery may outweigh the theoretical fire risk [4, 14]. The notion that environmental oxygen accumulation is unlikely to pose a major hazard is further supported by studies evaluating the safety of the pneumatically driven mechanical CPR device Lund University Cardiopulmonary Assist System (LUCAS I). In these investigations, oxygen was used as the driving gas, resulting in a continuous outflow of approximately 70 L/min directed toward the chest. Even under such circumstances, oxygen concentrations measured on the mannequin’s chest reached about 24% to 34–36% [6, 9].

Although oxygen concentrations above approximately 23.5–24% are commonly used as a precautionary threshold for identifying an oxygen-enriched atmosphere, this threshold does not indicate an abrupt transition to rapid combustion. For materials such as cotton, markedly accelerated flame propagation has been reported primarily at concentrations of approximately 30–35% and above [8]. Notably, in our study, the highest oxygen concentrations measured at the subclavian and axillary sites were 23.5% and 24.0%, respectively, remaining at or below the precautionary threshold and well below the range associated with markedly accelerated combustion.

Additionally, the effectiveness of removing oxygen sources as a preventive strategy is questionable. In the present study, oxygen concentrations neither showed a clinically relevant increase nor demonstrated any measurable decline after removal of the oxygen source prior to defibrillation. Similarly, experimental data from Greco et al., demonstrated that elevated oxygen concentrations may persist for 30–60 s after discontinuation of oxygen flow [10]. In time-critical resuscitation scenarios, this suggests that removing oxygen sources immediately prior to defibrillation may neither reliably nor rapidly reduce local oxygen concentrations. At the same time, such actions may delay defibrillation and disrupt ongoing oxygen delivery, thereby creating a potential conflict between theoretical fire prevention and the highest possible oxygenation of the patient.

The actual mechanisms underlying defibrillation-associated fires also warrant careful consideration [12, 17, 23]. The existing evidence is largely limited to a small number of case reports, most of which share common features, including the use of manual paddles, inappropriate electrode size, or suboptimal contact with the patient’s skin. These factors are known to increase the likelihood of electrical arcing. In contrast, to our knowledge, no cases of defibrillation-associated fires have been reported when using modern adhesive electrode pads. Consequently, preventive strategies of electrical arching might be more effectively directed toward ensuring proper electrode selection, positioning, and skin contact. A substantial part of the current understanding of fire risk during resuscitation seems to be based on data from operating room environments. In surgical environments, elevated fire risks are associated with the presence of flammable materials (e.g., alcohol-based disinfectants, surgical drapes), the frequent use of ignition sources such as electrocautery, and the proximity of oxygen delivery systems and the surgical field [3, 7, 11, 13, 16]. In contrast, during resuscitation, these conditions are typically absent or markedly less pronounced. Therefore, the transferability of fire risk considerations from other medical settings, particularly the operating room, to cardiopulmonary resuscitation is limited.

**Fig. 5:**
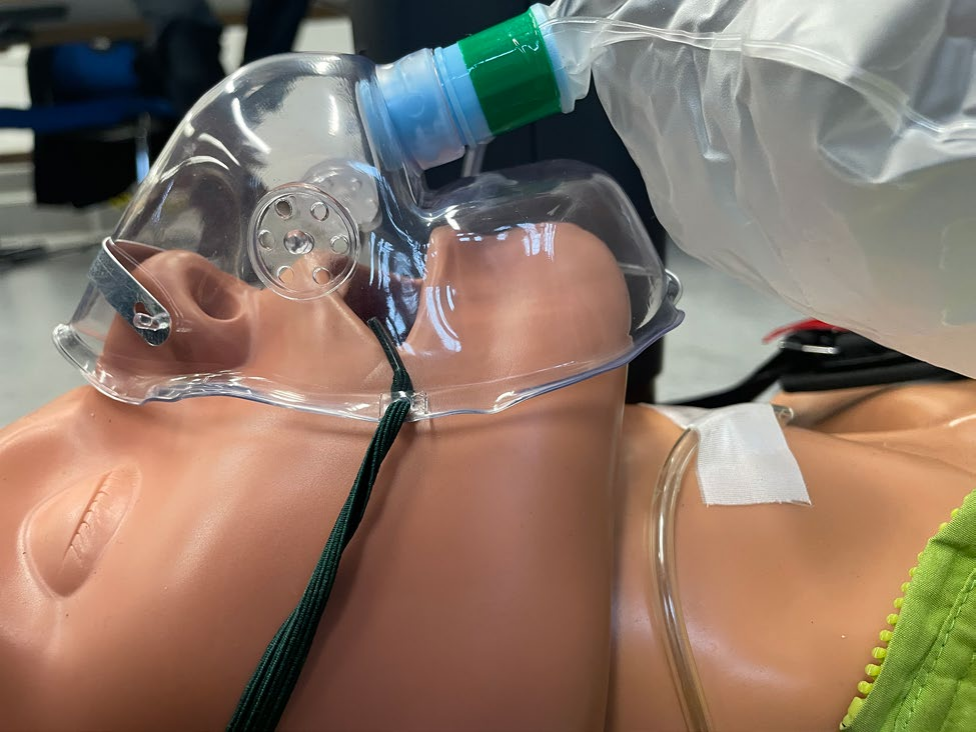
Example of oxygen leakage during use of a rebreathing mask: This snapshot illustrates a scenario evaluating oxygen concentrations during use of an oxygen rebreathing mask. Inadequate mask fit may create a gap at the chin, allowing oxygen to escape toward the neck. Despite this localised effect, oxygen concentrations in the chest region remained below 24%

Even though there were lower oxygen fractions reported when using an oxygen demand valve (OXYMAND, Weinmann, Germany) and when the airway was secured by a laryngeal mask or endotracheal tube, the effect size was minimal and unlikely to be clinically relevant [15]. The only scenario associated with a moderately increased oxygen concentration (up to 30%) was a poorly fitting oxygen rebreathing mask with significant leakage directed toward the neck region (Fig. 5). Importantly, even in this situation, oxygen concentrations at the electrode placement sites remained mainly unaffected. This observation underscores the importance of proper oxygen mask positioning and avoiding unnecessary manipulation, which may inadvertently increase leakage.

Several limitations must be acknowledged. First, this study was conducted using a manikin model and, therefore, cannot fully replicate the complexity of real-life resuscitation scenarios.

While the experiments were deliberately conducted without active ventilation and with only two rescuers to minimise externally induced air movement and thereby create conditions explicitly favourable to local oxygen accumulation, the model did not reproduce patient-specific factors such as a compliant and dynamically moving chest wall, body heat, perspiration, moisture from the airway, individual anatomy, or variable leakage around oxygen-delivery devices. In clinical settings, these factors may alter local airflow and oxygen dispersion as well as accumulation. On the other hand, in actual resuscitation settings, environmental airflow would commonly increase turbulence and may enhance oxygen dispersion. Second, measurements were limited to predefined locations and did not capture the full spatial distribution of oxygen concentrations or dynamic airflow patterns. In addition, the oxygen sensors used in this study had an upper measurement limit of 30%, precluding quantification of concentrations above this threshold. However, as only 19 of 6,555 measurements reached this upper limit and all other values remained below 30%, a relevant underestimation of markedly higher oxygen concentrations appears unlikely. Third, no direct assessment of ignition or combustion events was conducted, so conclusions regarding fire risk are indirect. Furthermore, the order of scenarios and repeated runs was not randomised or counterbalanced. Although the experimental area was ventilated between scenarios and each subsequent run was initiated only after ambient oxygen concentrations had returned to baseline, potential order effects, including sensor drift, changes in ambient conditions, or learning and fatigue effects among the resuscitation providers, cannot be entirely excluded. Finally, although efforts were made to simulate unfavourable conditions, real-world variability in environmental factors cannot be entirely excluded.

Despite these limitations, the present findings suggest that clinically relevant oxygen accumulation at defibrillation sites is unlikely under the tested conditions. Accordingly, routine removal of oxygen sources before defibrillation may not be necessary in many situations. Greater emphasis may instead be placed on proper placement of oxygen-delivery devices and minimisation of avoidable leakage when adjusting oxygen rebreathing masks and bag-valve-mask-ventilation. In addition, established risk factors for electrical arcing should be adressed, including the use of appropriately sized adhesive electrodes and ensuring optimal skin contact without folds or gaps. A more differentiated approach may therefore be warranted in future recommendations.

In conclusion, oxygen accumulation at clinically relevant sites during cardiopulmonary resuscitation appears to be limited. Likewise, removing oxygen-delivery devices in preparation for defibrillation did not result in a reduction in oxygen concentrations. Preventive strategies should prioritise avoiding electrical arcing over the routine removal of oxygen sources. Further studies in real-world settings are needed to confirm these findings and to inform evidence-based guideline development.

## Supplementary Information

Below is the link to the electronic supplementary material.

Supplementary Material 1: Supplement A: Time-oxygen concentration profiles of all scenarios

Supplementary Material 2: Supplement B: Measurements from a run with a tear in the artificial lung

## Data Availability

The data that support the findings of this scientific publication are available from the corresponding author upon reasonable request.

## Abbreviations

ALS: Advanced life support
BLS: Basic life support
CPR: Cardio-pulmonary resuscitation
ERC: European Resuscitation Council
LUCAS: Lund University Cardiopulmonary Assist System
mCPR: Mechanical cardio-pulmonary resuscitation

## References

[1] Ambu GmbH. n.d. Ambu^®^ Man Basic Datenblatt. Ambu^®^ Man Basic Datenblatt. Accessed August 23, 2025. https://www.ambu.de/Admin/Public/DWSDownload.aspx?File=%2fFiles%2fFiles%2fDownloads%2fAmbu+DE%2fEmergency_Care%2fTrainings_Manikins_BLS%2fAmbu+Man+Basic%2fDatenblatt%2fDB_AmbuMan_Basic_493518522_V05_042021.pdf

[2] AmbuMan^®^. Airway Instrument. n.d. Accessed April 10, 2026. https://www.ambu.de/notfallmedizin-reanimationspuppen/als-trainingsgerate/produkt/ambuman-airway-instrument-1.

[3] Barker SJ, Polson JS. Fire in the Operating Room: A Case Report and Laboratory Study. Anesth Analg. 2001;93(4):960–5. 11574364 10.1097/00000539-200110000-00031

[4] Cantello E, Davy TE, Koenig KL. The Question of Removing a Ventilation Bag before Defibrillation. J Accid Emerg Med. 1998;15(4):286. 9681318 10.1136/emj.15.4.286 PMC1343148

[5] Daly S, Milne HJ, Holmes DP, and Alasdair R. Corfield. Defibrillation and External Pacing in Flight: Incidence and Implications. Emerg Med J. 2014;31(1):69–71. 23264607 10.1136/emermed-2012-202028

[6] Deakin CD, Paul V, Fall E, Petley GW, and Fizz Thompson. Ambient Oxygen Concentrations Resulting from Use of the Lund University Cardiopulmonary Assist System (LUCAS) Device during Simulated Cardiopulmonary Resuscitation. Resuscitation. 2007;74(2):303–9. 17507141 10.1016/j.resuscitation.2007.04.001

[7] Engel SJ, Nikesh K, Patel CM, Morrison, et al. Operating Room Fires: Part II. Optimizing Safety: Part II. Optimizing Safety. Plast Reconstr Surg. 2012;130(3):681–9. 22575855 10.1097/PRS.0b013e31825dc14a

[8] European Industrial Gases Association. Fire hazards of oxygen and oxygen-enriched atmospheres. European Industrial Gases Association; 2018. https://www.eiga.eu/uploads/documents/DOC004.pdf.

[9] Farmery JS, Carter M. Use of the Lund University Cardiopulmonary Assist System in the MD902: A Fire Safety Assessment. Emerg Med J. 2007;24(2):110–1. 17251616 10.1136/emj.2006.039768 PMC2658184

[10] Greco RJ, Gonzalez R, Johnson P, Scolieri M, Rekhopf PG, Heckler F. Potential Dangers of Oxygen Supplementation during Facial Surgery. Plast Reconstr Surg. 1995;95(6):978–84. 7732145 10.1097/00006534-199505000-00004

[11] Huddleston S, Hamadani S, Phillips ME, and James C. Fleming. Fire Risk during Ophthalmic Plastic Surgery. Ophthalmology. 2013;120(6):1309e1. 10.1016/j.ophtha.2013.01.016 23732059

[12] Hummel RS. Spark-Generating Properties of Electrode Gels Used during Defibrillation: A Potential Fire Hazard. JAMA: J Am Med Association. 1988;260(20):3021. 3184368

[13] Kezze I, Zoremba N, Rossaint R, Rieg A, Coburn M, Schälte G. Risks and Prevention of Surgical Fires: A Systematic Review: A Systematic Review. Anaesthesist. 2018;67(6):426–47. Originally published as Risiken Und Prävention Chirurgisch Induzierter Feuer: Eine Systematische Übersicht. 29766207 10.1007/s00101-018-0445-2

[14] McAnulty GR, Robertshaw H. Risk of Fire Outweighed by Need for Oxygen and Defibrillation. J Accid Emerg Med. 1999;16(1):77. 9918303 10.1136/emj.16.1.77-c PMC1343276

[15] Meier-Medizintechnik GmbH. n.d. Weinmann OXYMAND Demandventil mit Stecker Typ Walther und Adapter COMBIBAG. Accessed April 11, 2026. https://www.meier-medizintechnik.de/weinmann-oxymand-demandventil-mit-stecker-typ-walther-und-adapter-combibag/wm-22128.

[16] Meneghetti S, Cristina MM, Morgan J, Fritz RG, Borkowski R, Djohan, Zins JE. Operating Room Fires: Optimizing Safety. Plast Reconstr Surg. 2007;120(6):1701–8. 18040210 10.1097/01.prs.0000282729.23202.da

[17] Miller PH. Potential Fire Hazard in Defibrillation. JAMA: J Am Med Association. 1972;221(2):192. 5067634

[18] Orhan-Sungur M, Komatsu R, Sherman A, Jones L, Walsh D, Sessler DI. Effect of Nasal Cannula Oxygen Administration on Oxygen Concentration at Facial and Adjacent Landmarks. Anaesthesia. 2009;64(5):521–6. 19413822 10.1111/j.1365-2044.2008.05820.x

[19] Oxygen use in the workplace. n.d. Accessed April 30. 2026. https://www.hse.gov.uk/pubns/indg459.htm.

[20] Robertshaw H, McAnulty G. Ambient Oxygen Concentrations during Simulated Cardiopulmonary Resuscitation. Anaesthesia. 1998;53(7):634–7. 9771170 10.1046/j.1365-2044.1998.410-az0512.x

[21] Soar J, Böttiger BW, Carli P, et al. European Resuscitation Council Guidelines 2021: Adult Advanced Life Support. Resuscitation. 2021;161(April):115–51. 33773825 10.1016/j.resuscitation.2021.02.010

[22] Soar J, Böttiger BW, Carli P, et al. European Resuscitation Council Guidelines 2025 Adult Advanced Life Support. Resuscitation. 2025;215(Suppl 1):110769. 41117572 10.1016/j.resuscitation.2025.110769

[23] Theodorou AA, Juan A, Gutierrez, Berg RA. Fire Attributable to a Defibrillation Attempt in a Neonate. Pediatrics. 2003;112(3 Pt 1):677–9. 12949302 10.1542/peds.112.3.677

