# Supplementary material for: Ambient oxygen enrichment during cardiopulmonary resuscitation: an overestimated risk? - Insights from a manikin study
Source: Scand J Trauma Resusc Emerg Med. 2026 Aug 11;34:133. doi: 10.1186/s13049-026-01669-3 (PMC13474727; doi:10.1186/s13049-026-01669-3)
Supplement: Supplementary file 1 [file 13049_2026_1669_MOESM1_ESM.pdf]

Ambient Oxygen-Enriched Atmospheres During Cardiopulmonary Resuscitation: An Overestimated Risk? -

Insights from a Manikin Study

Dominik J Hoechter; Florian Pfeiffer; Michael Storz; Andreas Higl; Bjarne Schmalbach;Katja Petrowski; Tobias Achtsnit

Supplement A

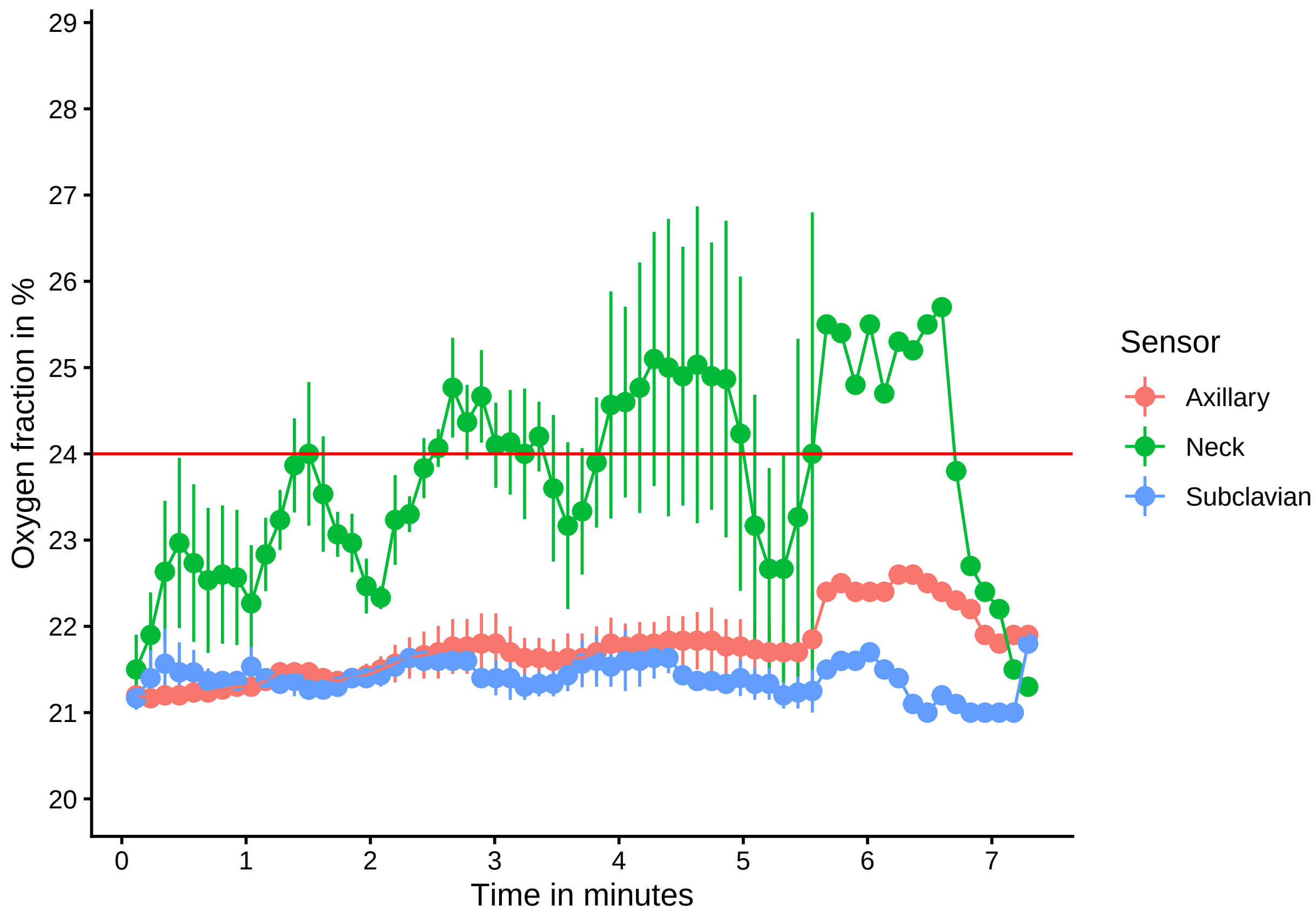
Oxygen fraction in %
29
28
27
26
25
24
23
22
21
20
0
1
2
3
4
5
6
7
Time in minutes
Sensor
Axillary
Neck
Subclavian

```
## [1] "Scenario 1"
```

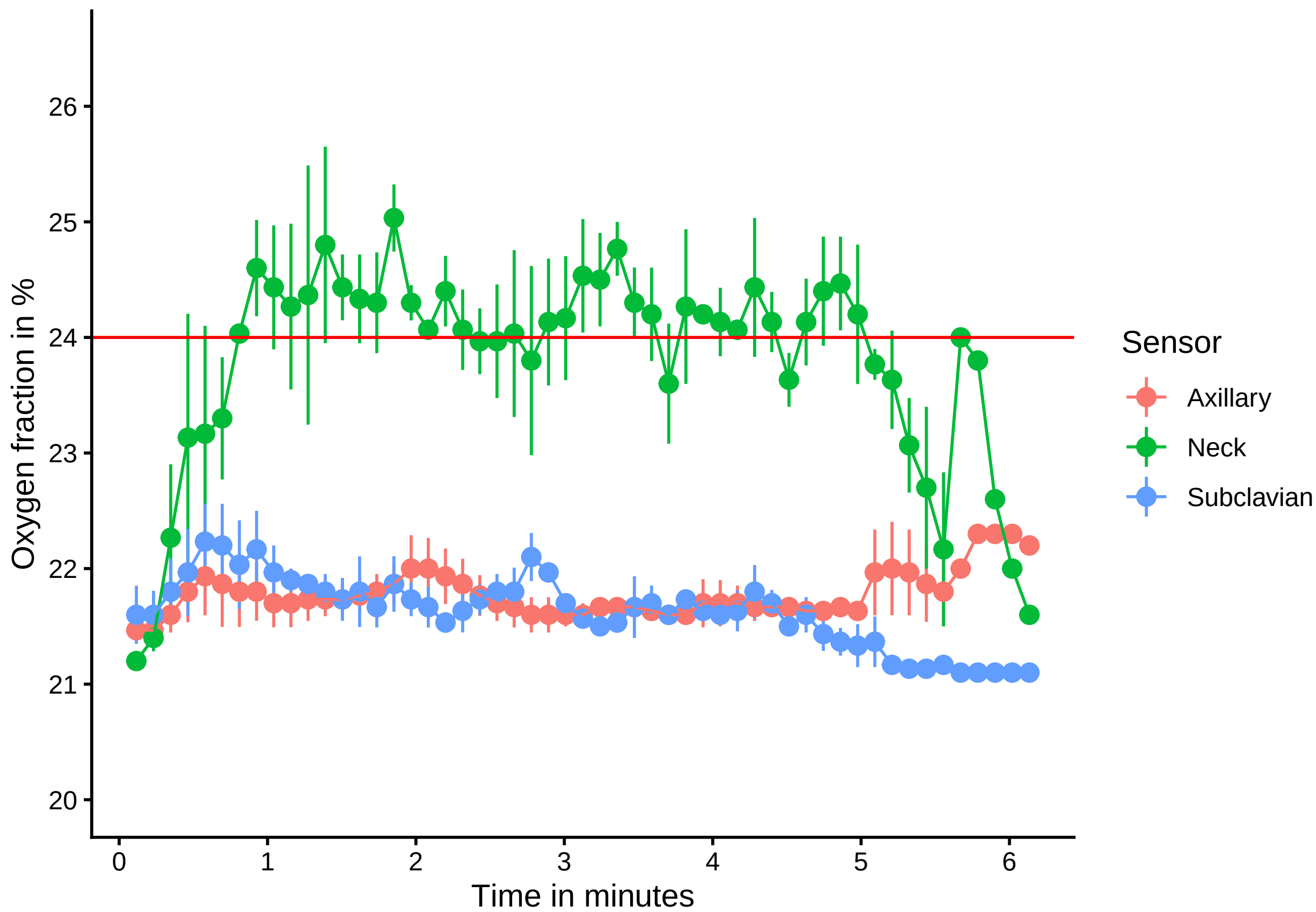

Oxygen fraction in %
26
25
24
23
22
21
20
0
1
2
3
4
5
6
Time in minutes
Sensor
Axillary
Neck
Subclavian


```
## [1] "Scenario 2"
```

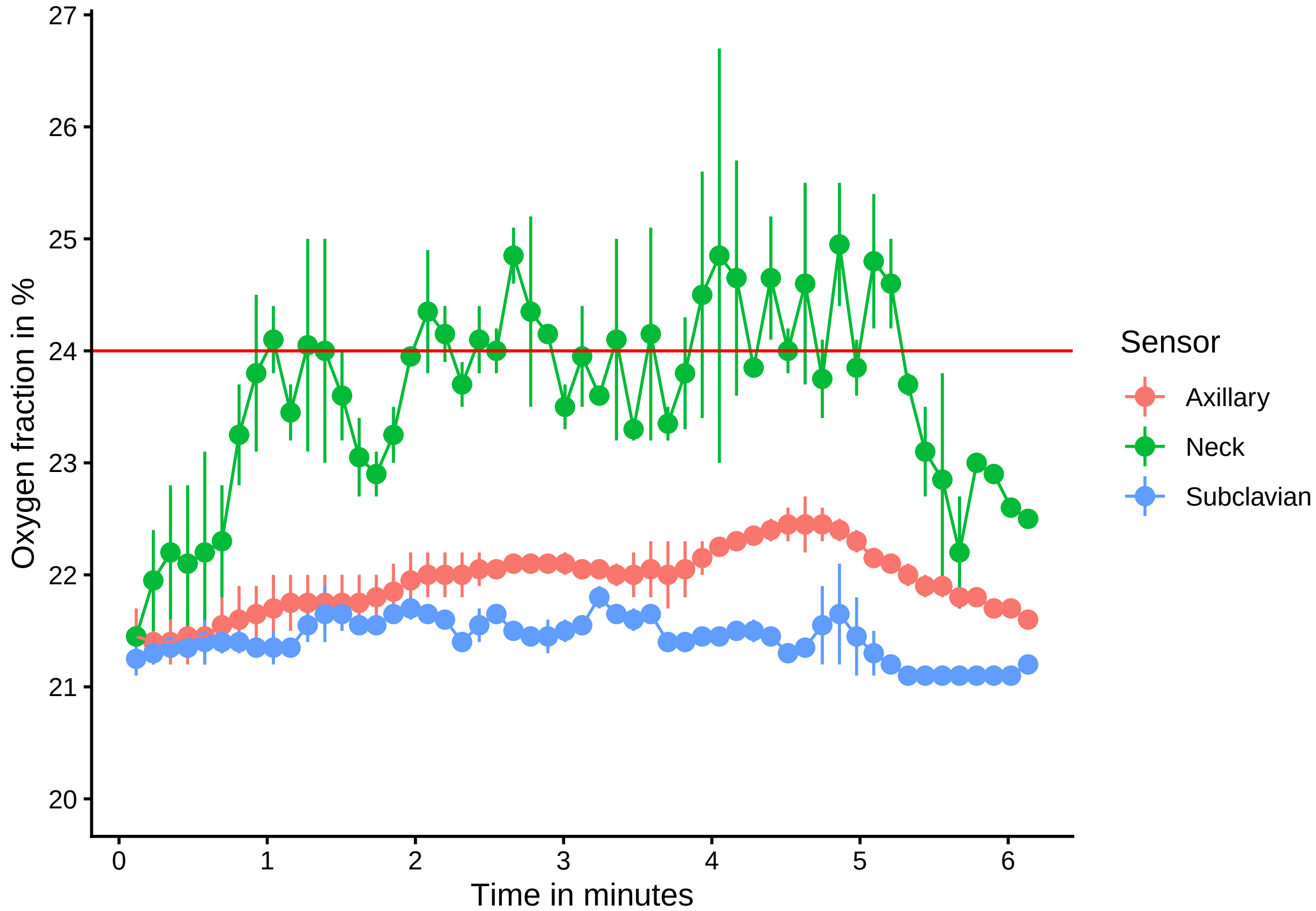

27
26
25
24
23
22
21
20
Oxygen fraction in %
0
1
2
3
4
5
6
Time in minutes
Sensor
Axillary
Neck
Subclavian


```
## [1] "Scenario 3"
```

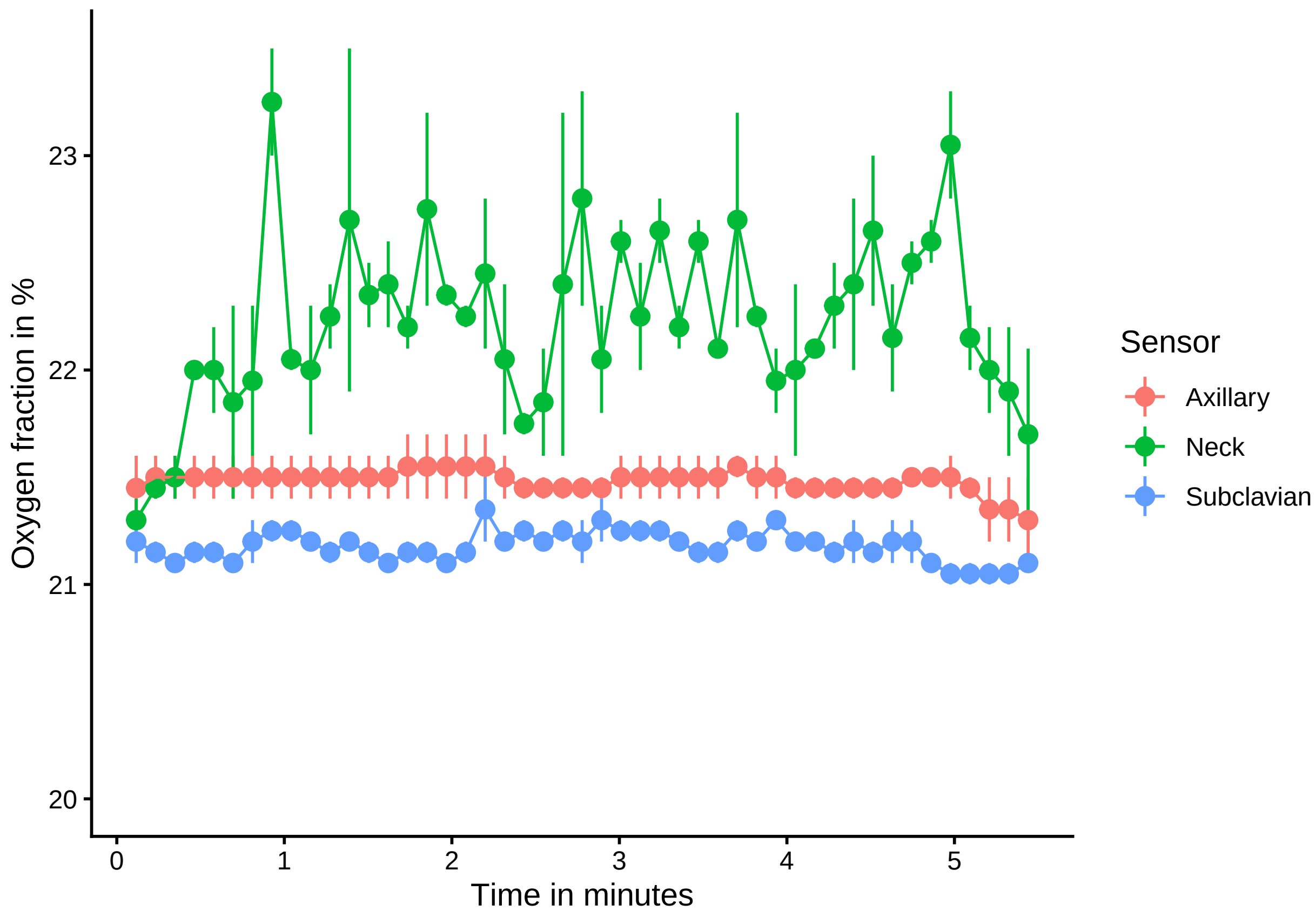
Oxygen fraction in %
20
21
22
23
0
1
2
3
4
5
Time in minutes
Sensor
Axillary
Neck
Subclavian

```
## [1] "Scenario 4"
```

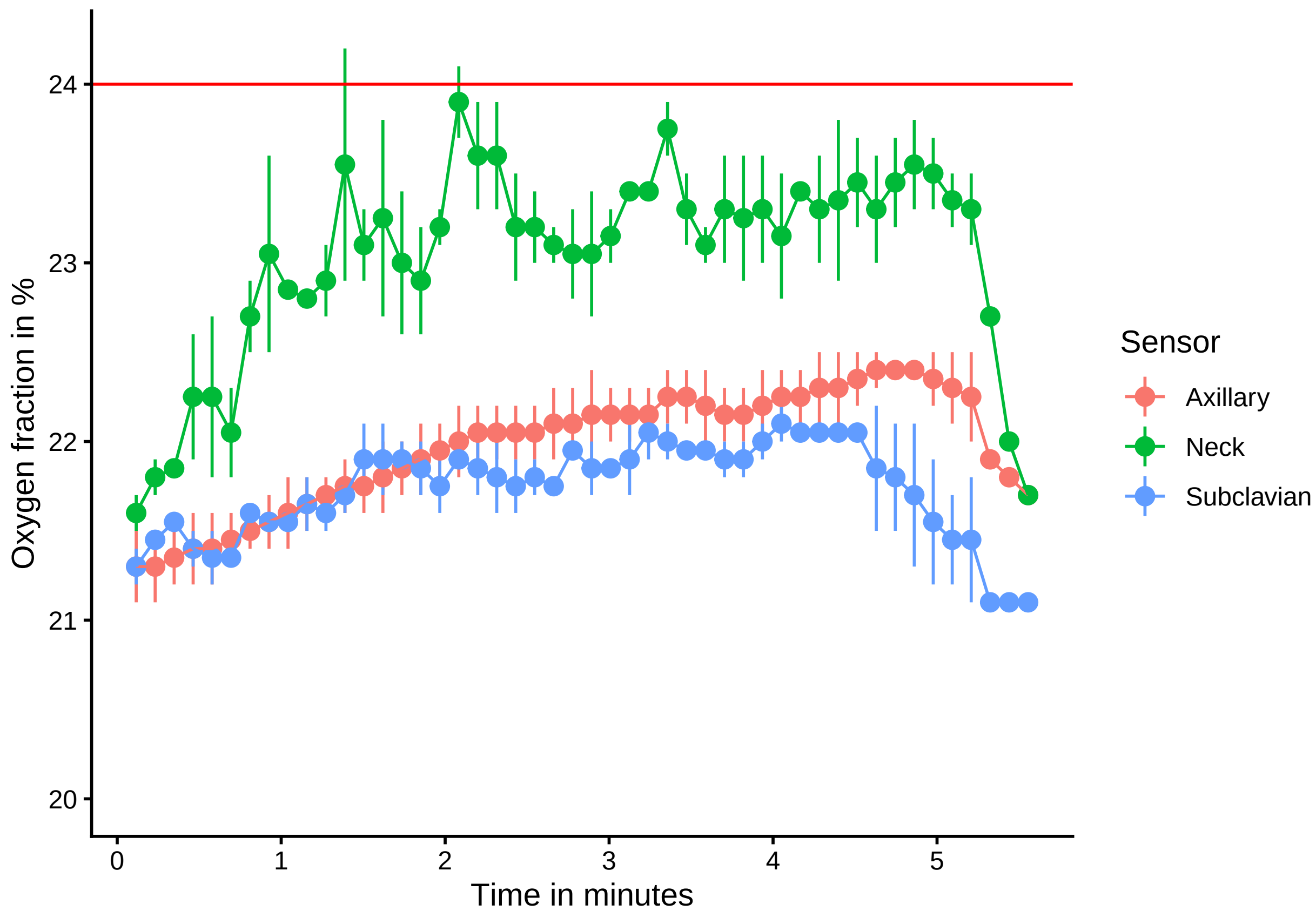

24
23
22
21
20
Oxygen fraction in %
0
1
2
3
4
5
Time in minutes
Sensor
Axillary
Neck
Subclavian


```
## [1] "Scenario 5"
```

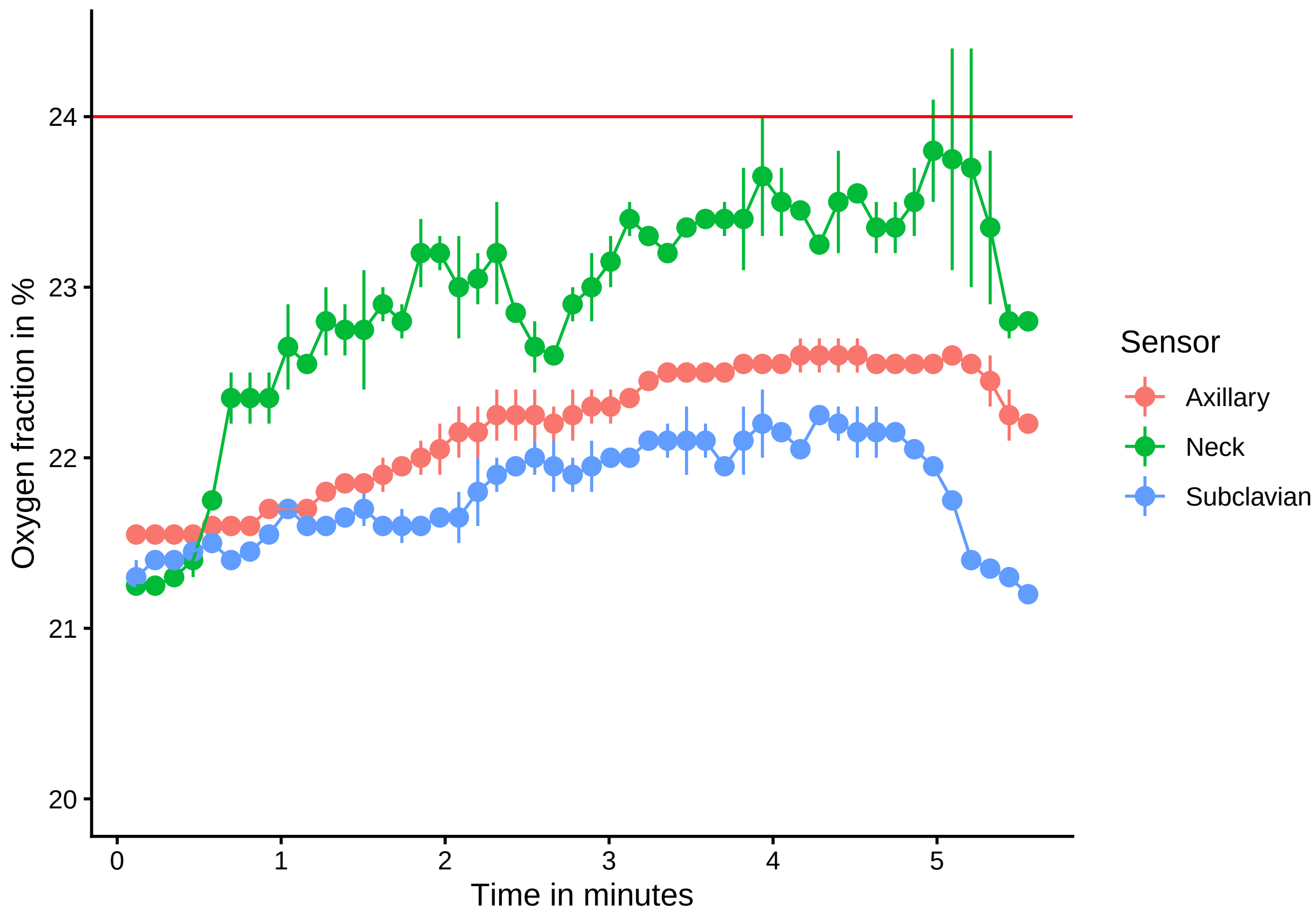

Oxygen fraction in %
24
23
22
21
20
0
1
2
3
4
5
Time in minutes
Sensor
Axillary
Neck
Subclavian


```
## [1] "Scenario 6"
```

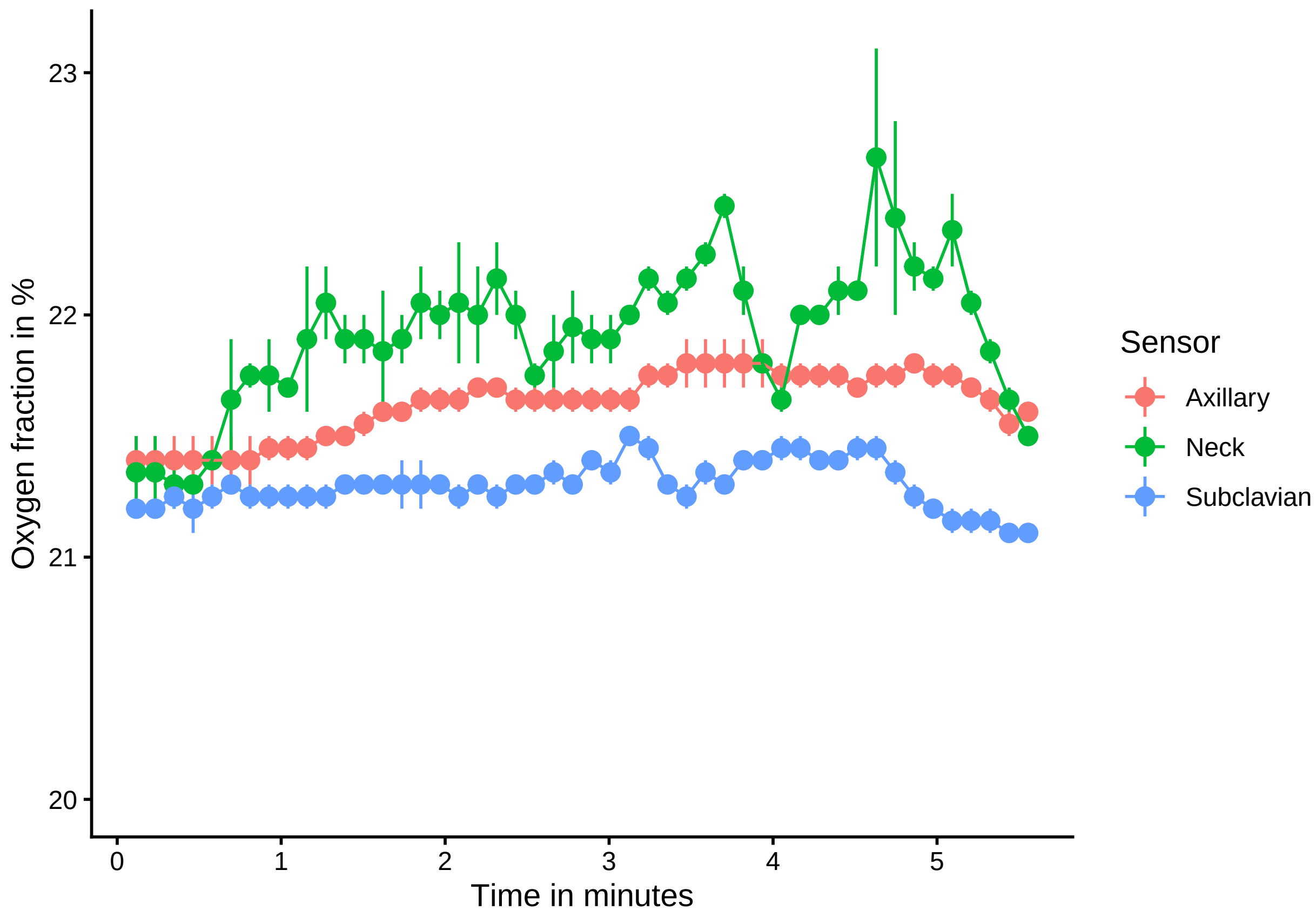

Oxygen fraction in %
23
22
21
20
0
1
2
3
4
5
Time in minutes
Sensor
Axillary
Neck
Subclavian


```
## [1] "Scenario 7"
```

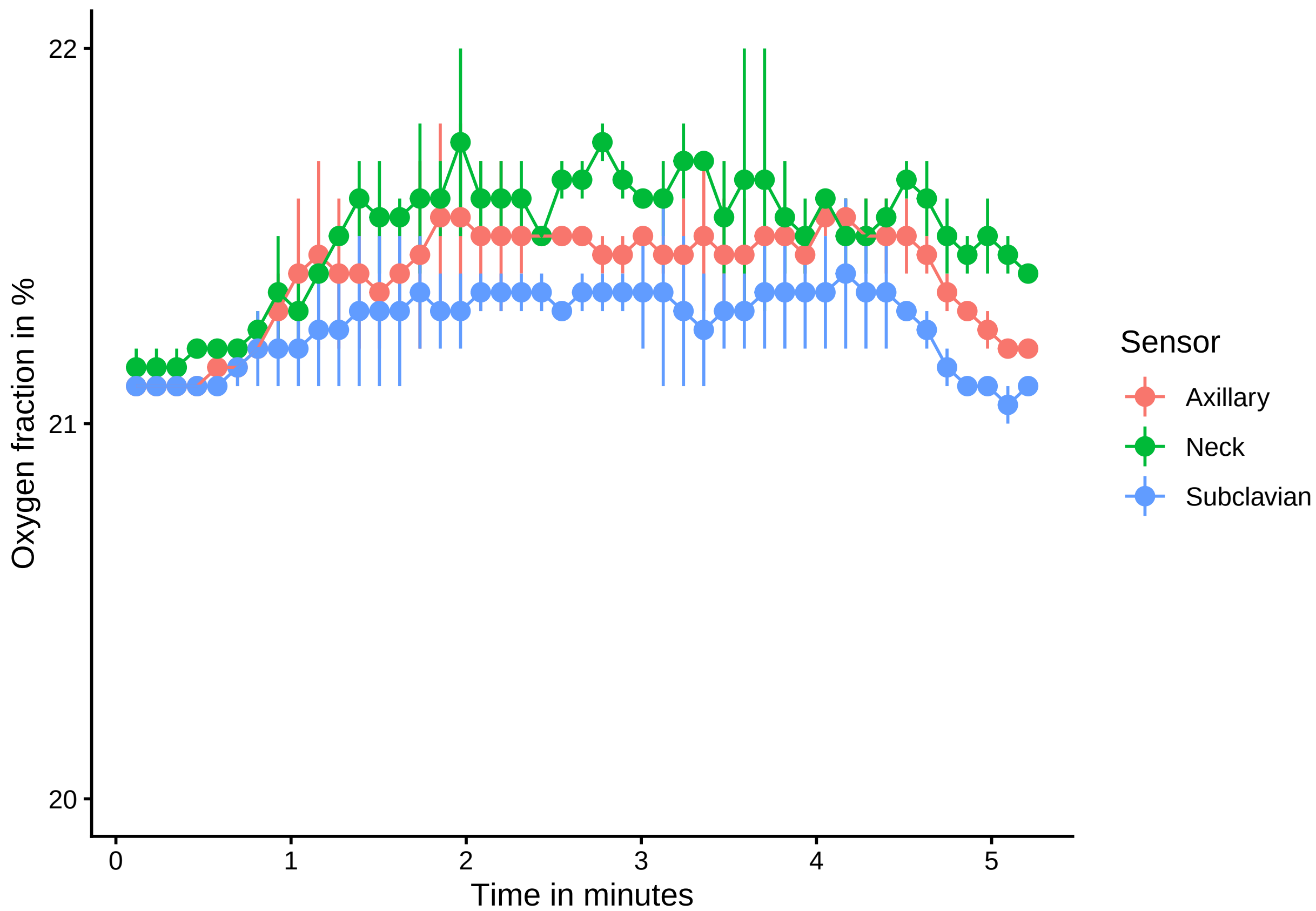
22
21
20
Oxygen fraction in %
0
1
2
3
4
5
Time in minutes
Sensor
Axillary
Neck
Subclavian

```
## [1] "Scenario 8"
```

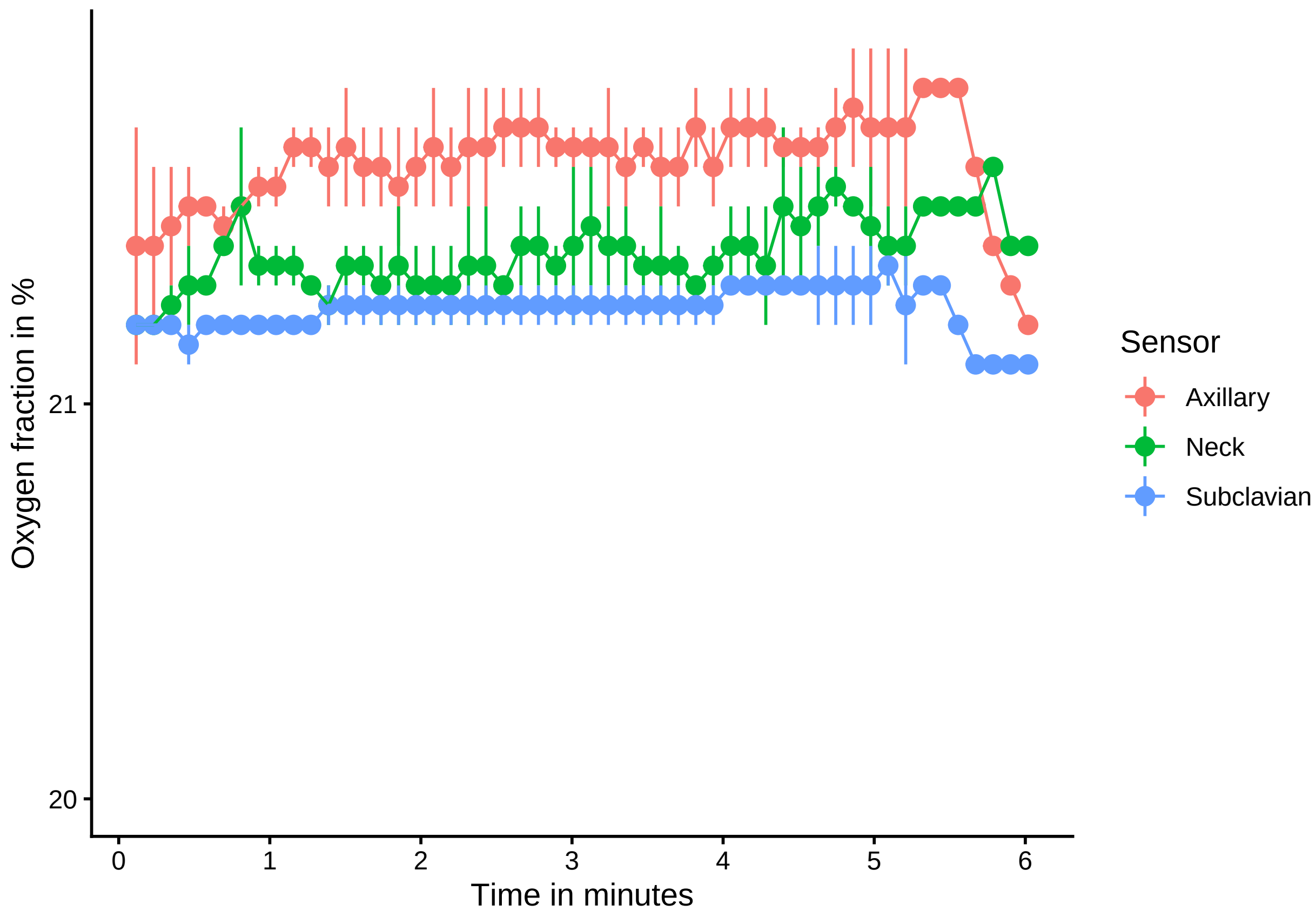

Oxygen fraction in %
21
20
0
1
2
3
4
5
6
Time in minutes
Sensor
Axillary
Neck
Subclavian


```
## [1] "Scenario 9"
```

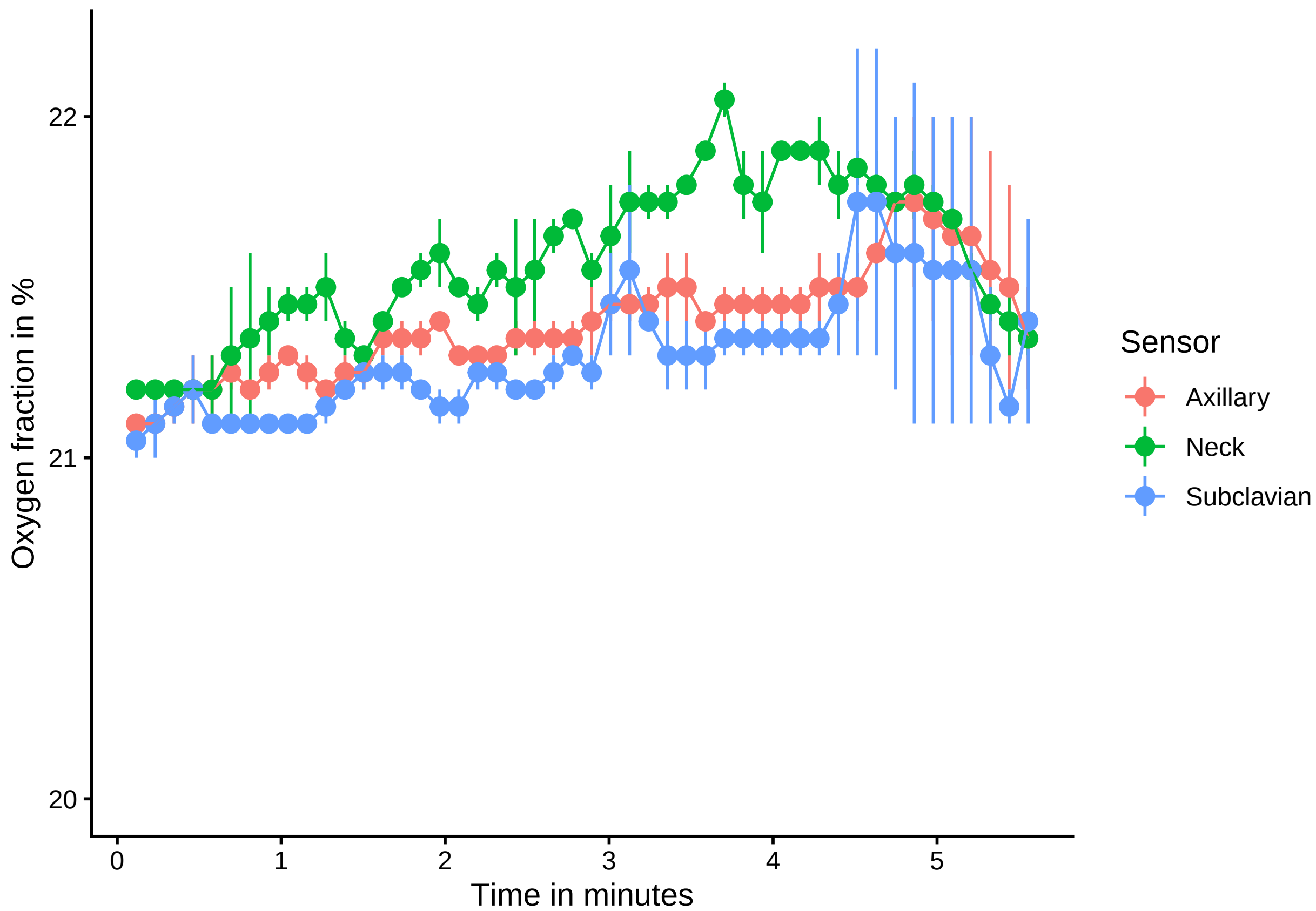


```
## [1] "Scenario 10"
```

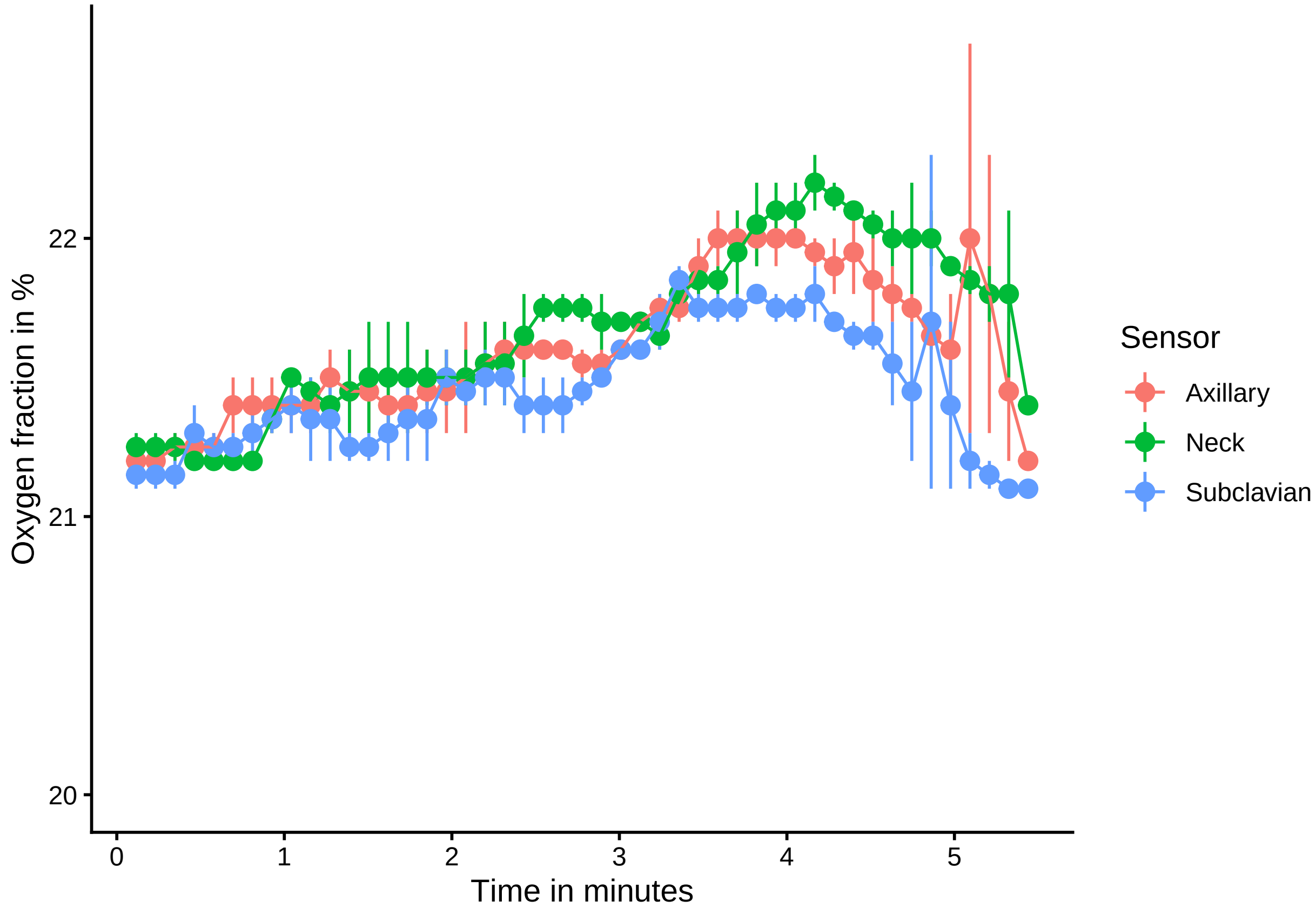


```
## [1] "Scenario 11"
```

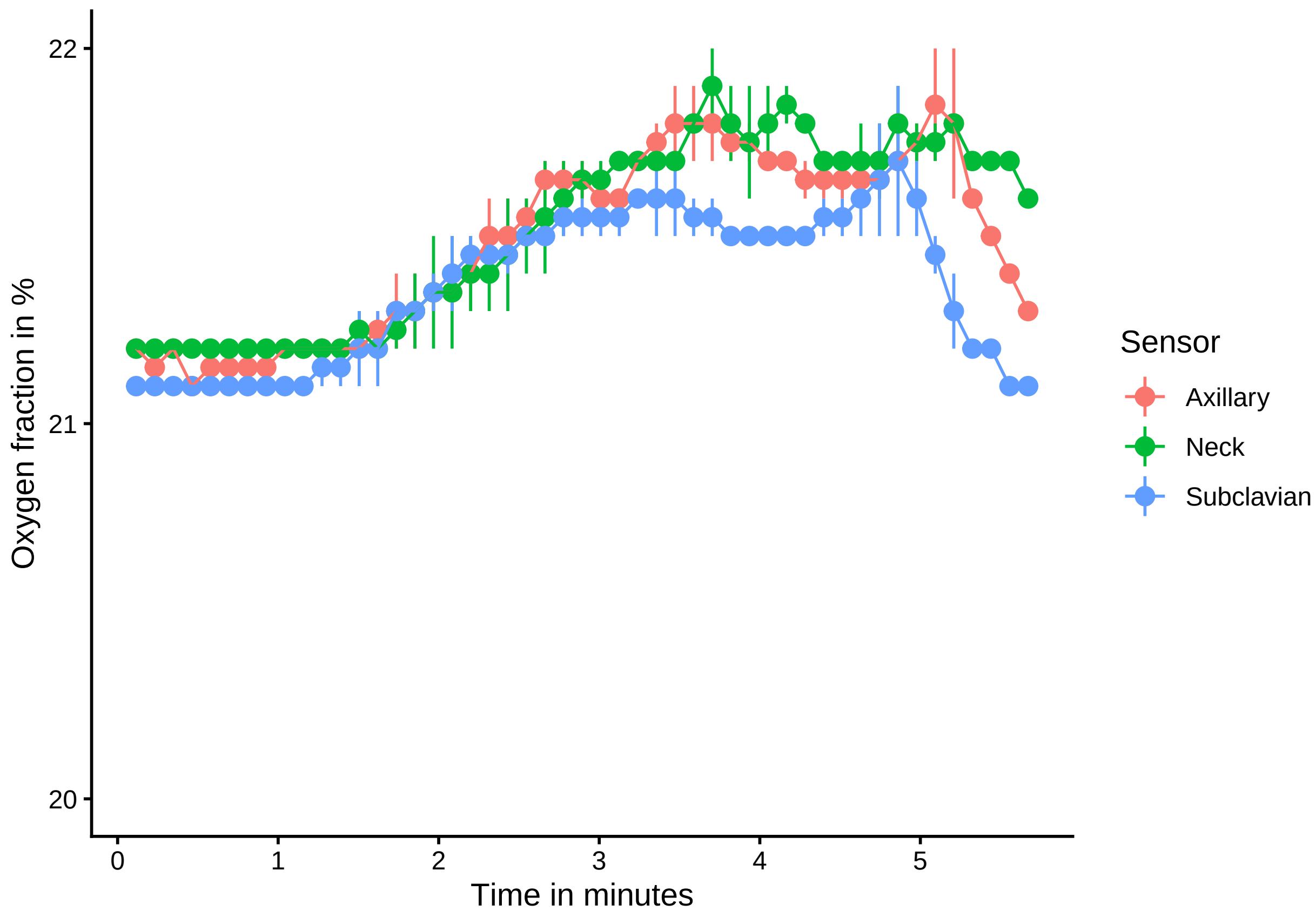


```
## [1] "Scenario 12"
```

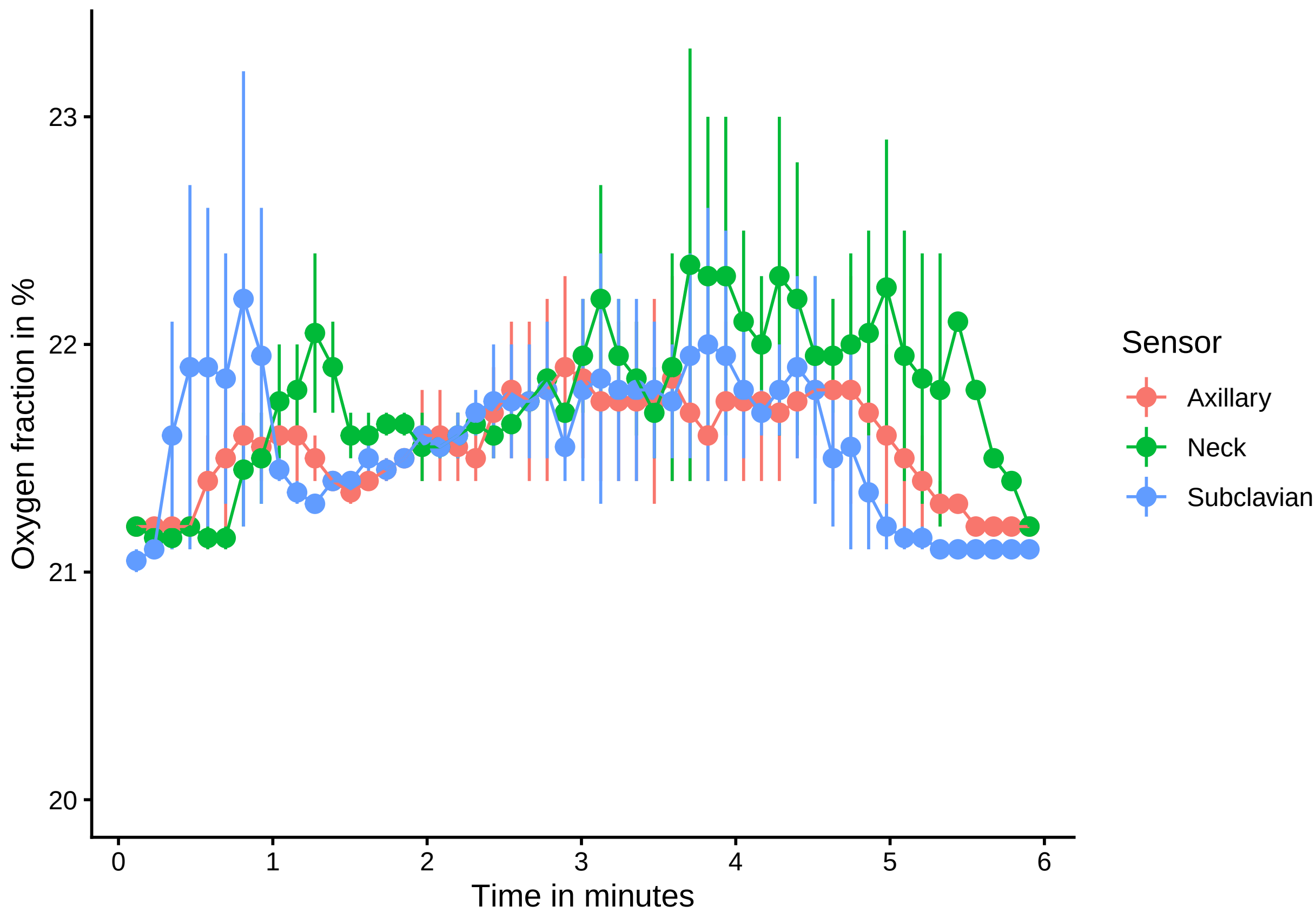


```
## [1] "Scenario 13"
```

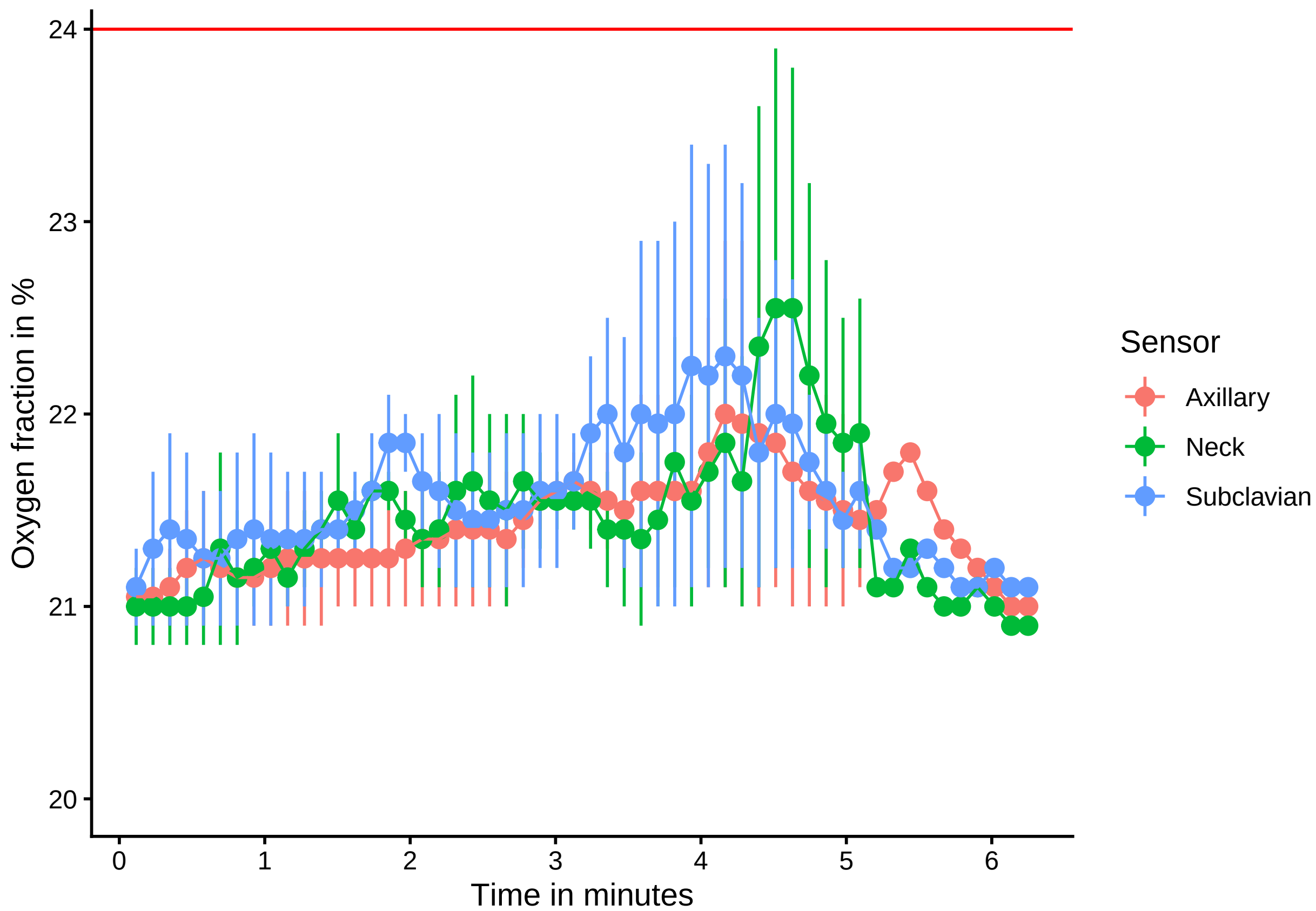


```
## [1] "Scenario 14"
```

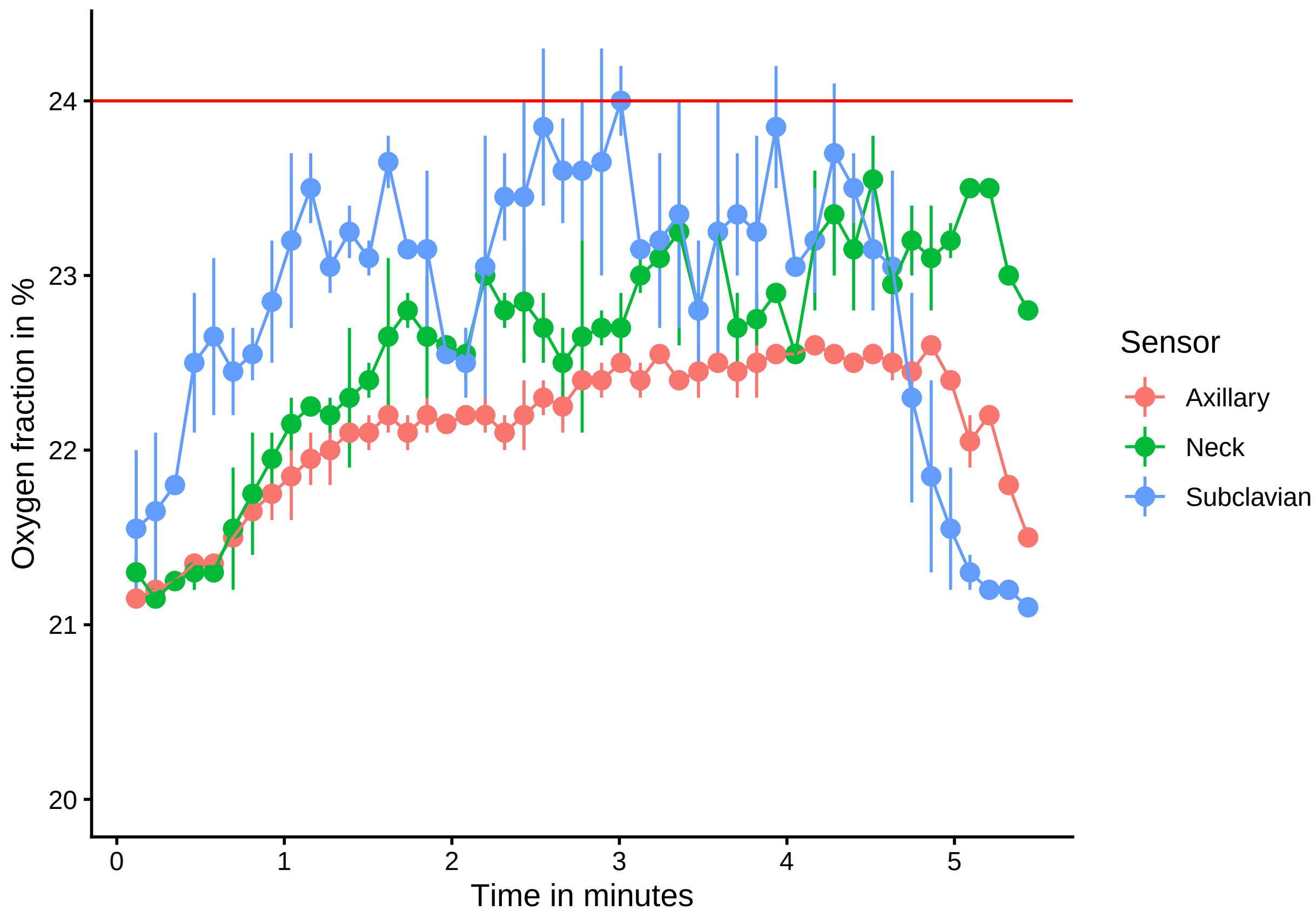


```
## [1] "Scenario 15"
```

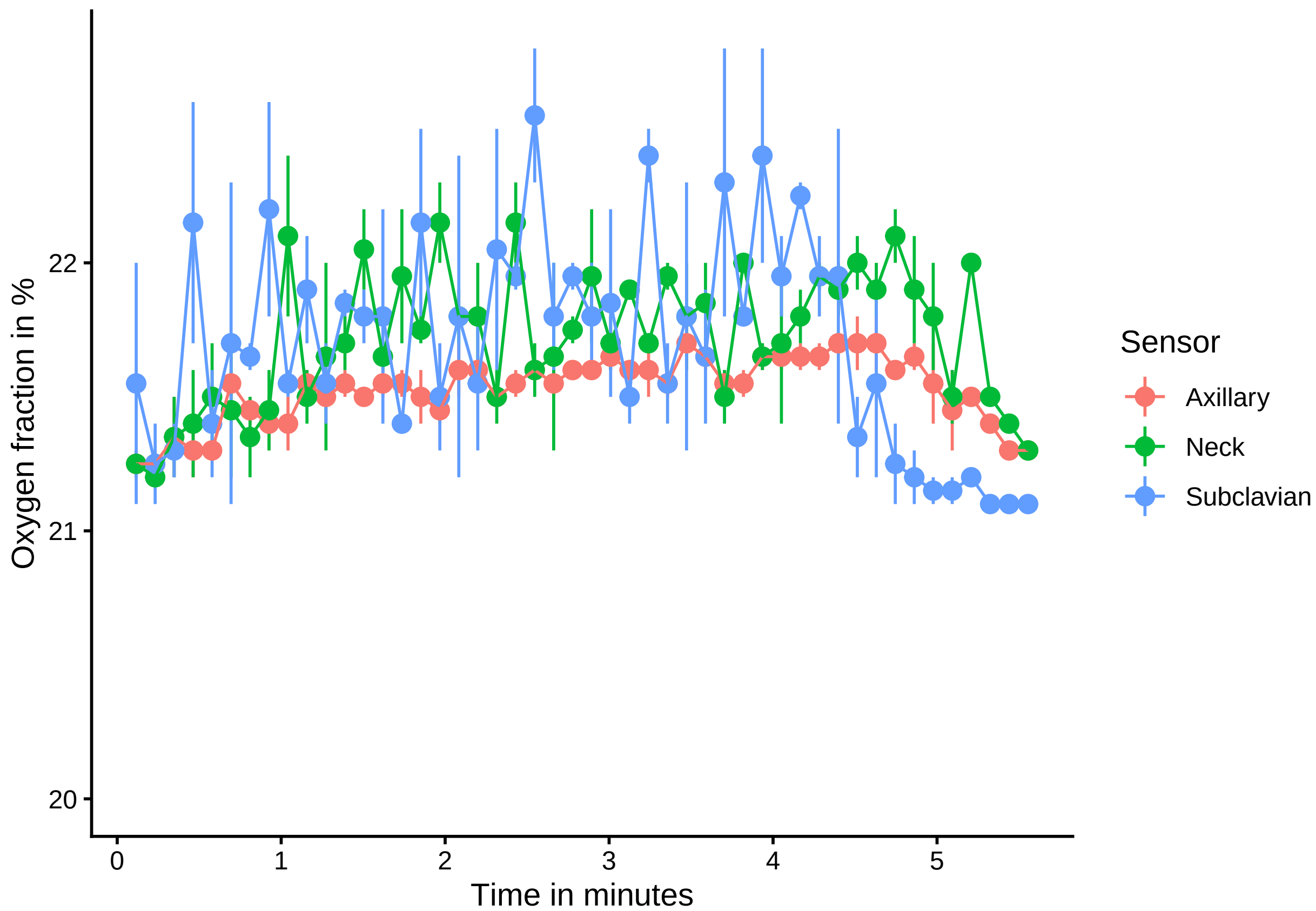


```
## [1] "Scenario 16"
```

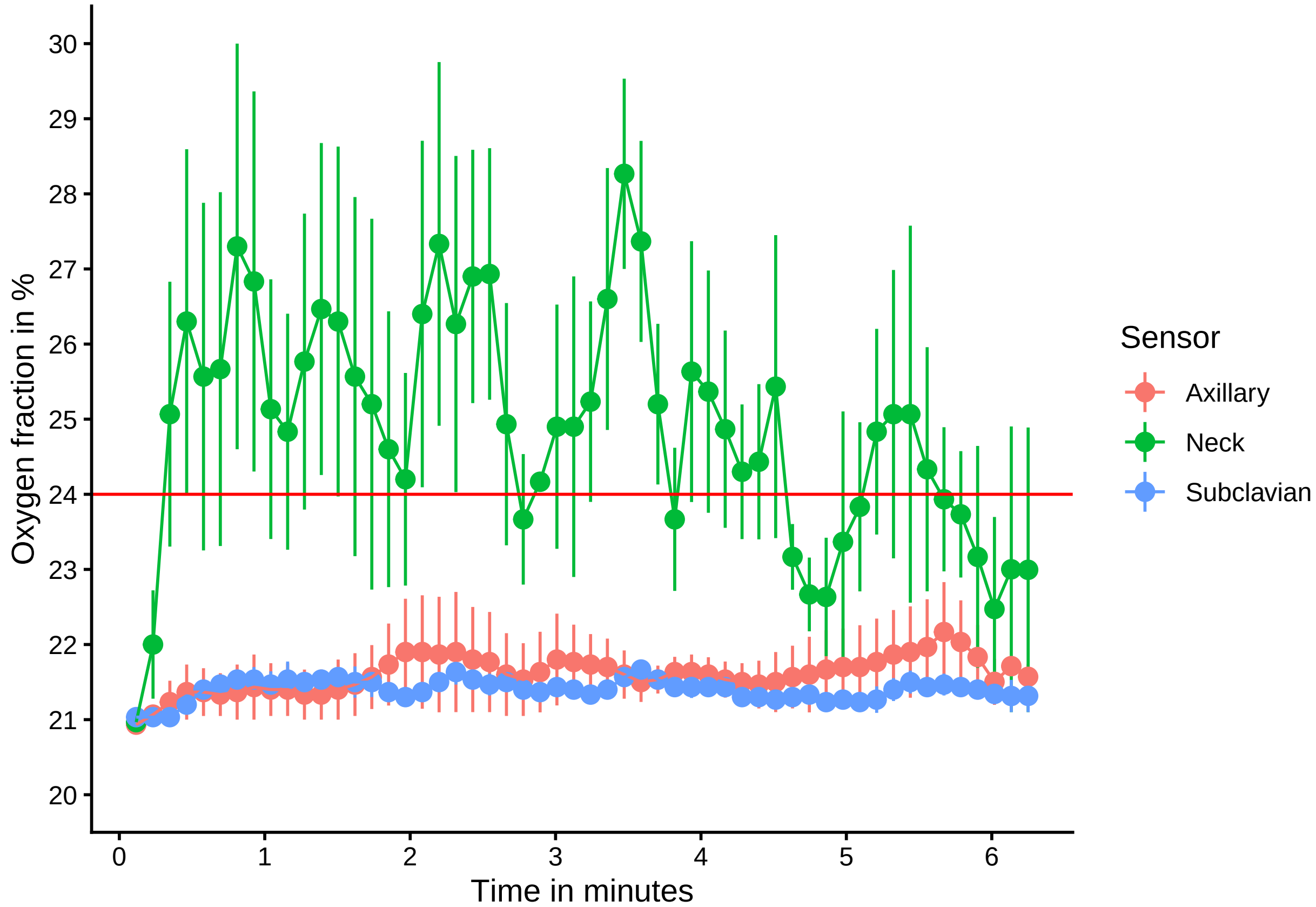


```
## [1] "Scenario 17"
```

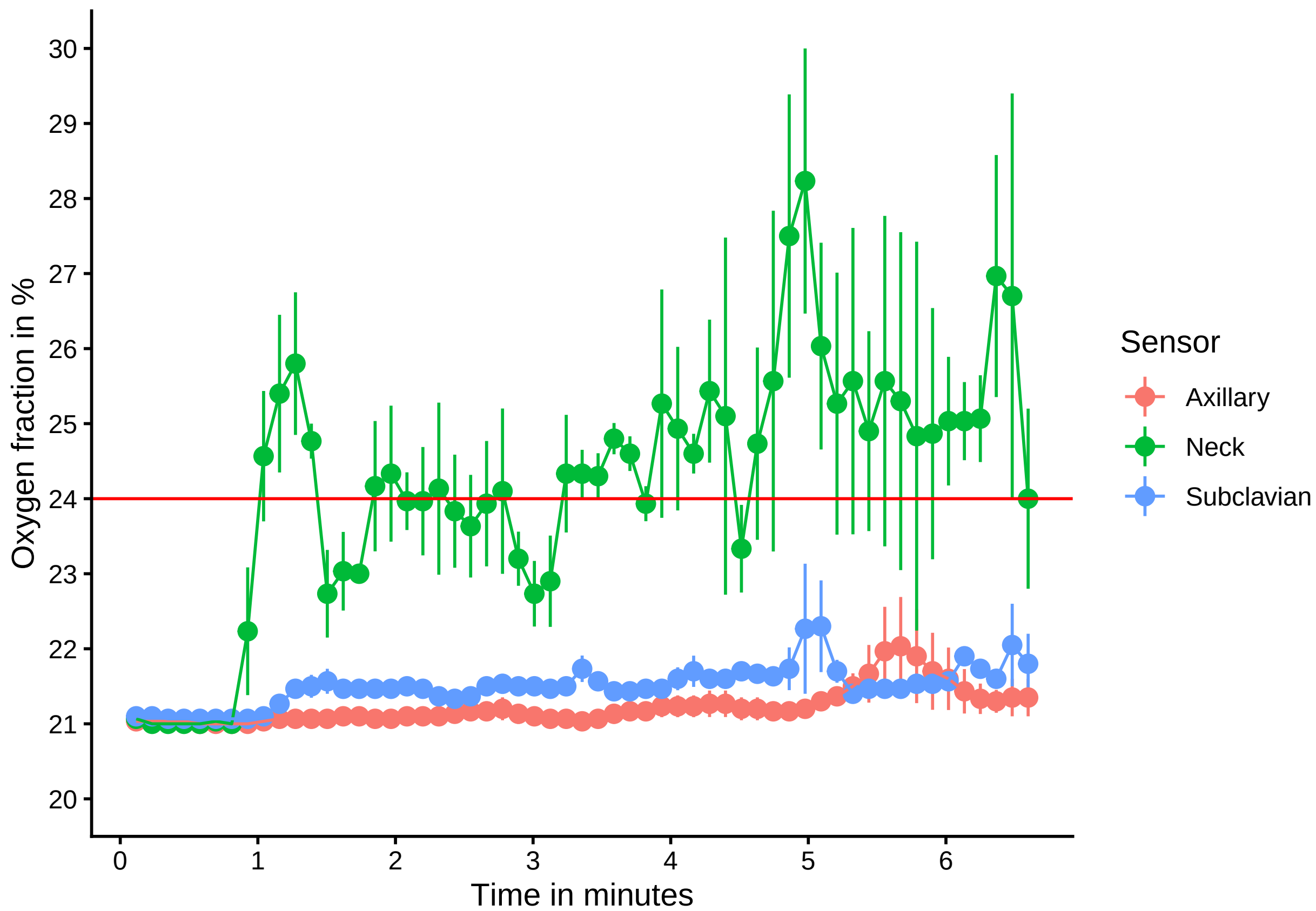


```
## [1] "Scenario 18"
```

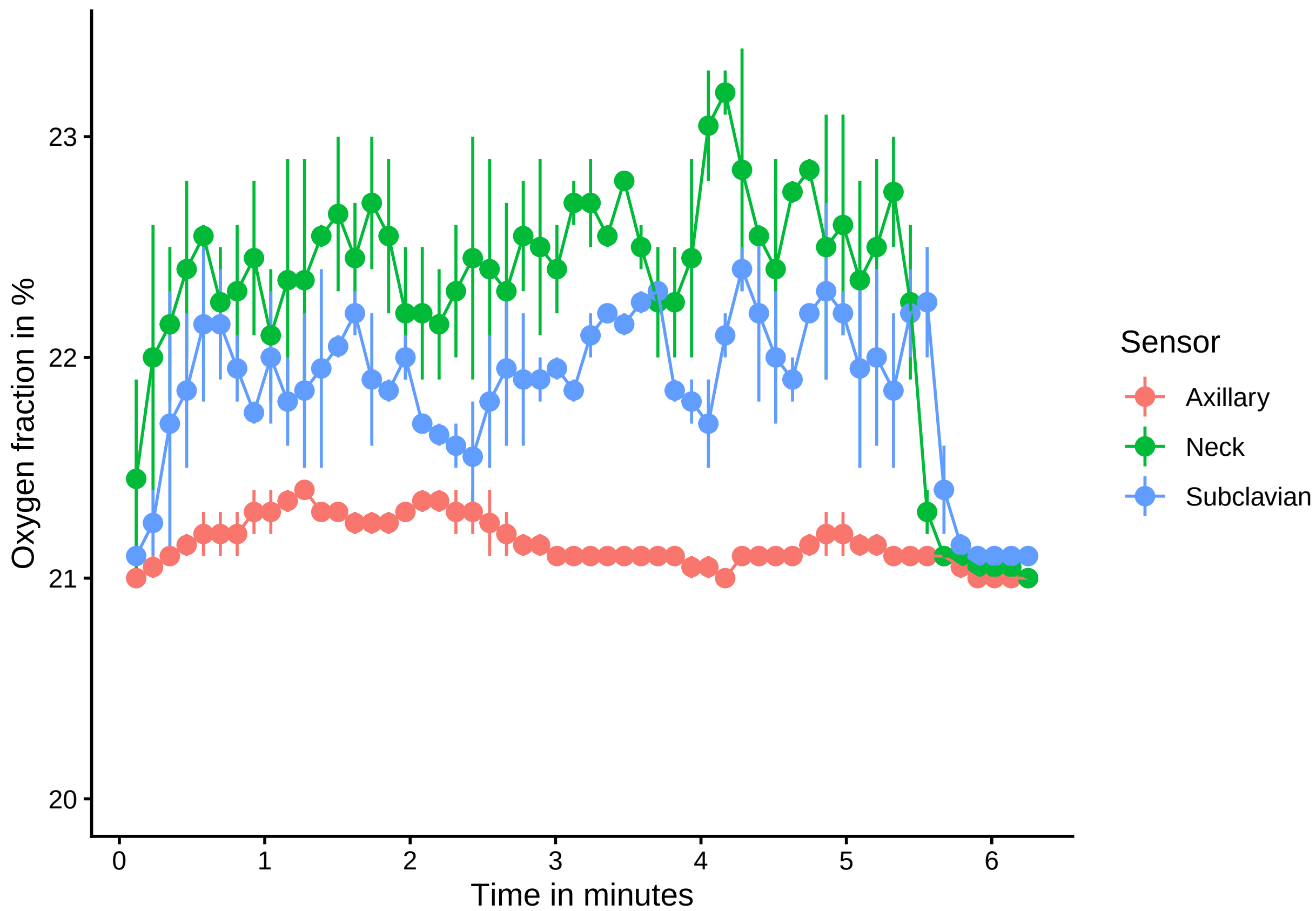


```
## [1] "Scenario 19"
```

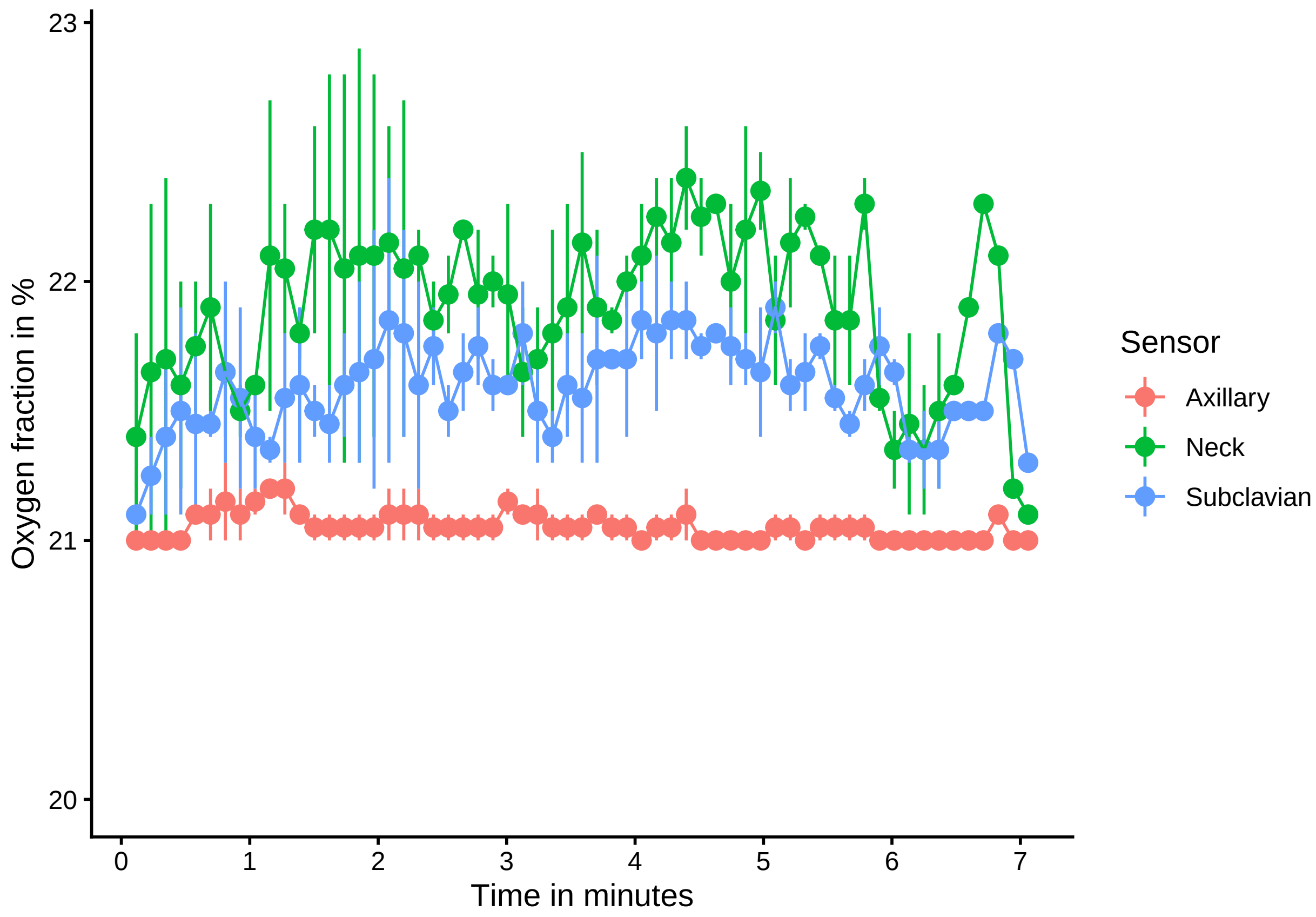


```
## [1] "Scenario 20"
```
