# Supplementary material for: Ambient oxygen enrichment during cardiopulmonary resuscitation: an overestimated risk? - Insights from a manikin study
Source: Scand J Trauma Resusc Emerg Med. 2026 Aug 11;34:133. doi: 10.1186/s13049-026-01669-3 (PMC13474727; doi:10.1186/s13049-026-01669-3)
Supplement: Supplementary file 2 [file 13049_2026_1669_MOESM2_ESM.pdf]

Ambient Oxygen Enrichment During Cardiopulmonary Resuscitation: An Overestimated Risk? - Insights from a Manikin Study

Dominik J Hoechter; Florian Pfeiffer; Michael Storz; Andreas Higl; Bjarne Schmalbach;Katja Petrowski; Tobias Achtsnit

Supplement B

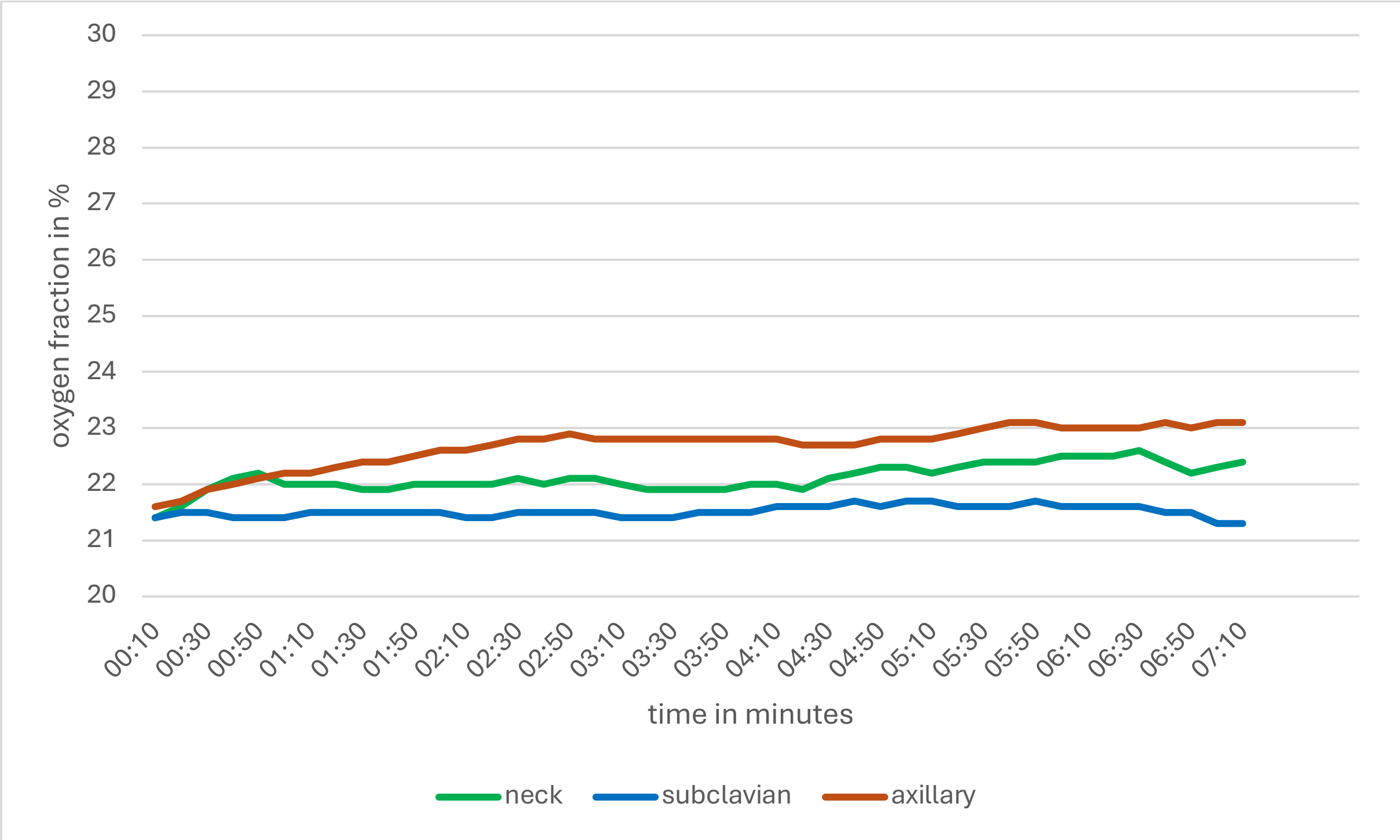

Measurements from a run with a tear in the artificial lung:
scenario 5: site: ambulance; airway: open; ventilation bag-valve-mask; oxygen: reservoir; upper garments: lifted; chest compressions: mCPR - LUCAS 3 ®; distance: <0,2m;

During the experiments, the oxygen monitor positioned in the manikin's left axillary region and a second monitor placed on the ambulance floor both triggered an "elevated oxygen concentration" alarm, whereas no increase was detected at the manikin's neck. Auscultation of the left hemithorax, followed by internal inspection of the manikin, revealed a hole or tear in the artificial lung. After replacement of the bag simulating the lung, the finding could no longer be reproduced.
